# Supplementary material for: Species-specific structural adaptation of the potyviral coat protein in virions and virus-like particles
Source: Commun Biol. 2026 Jan 13;9:226. doi: 10.1038/s42003-025-09502-w (PMC12902108; doi:10.1038/s42003-025-09502-w)
Supplement: Supplementary file 2 — Supplementary Information [file 42003_2025_9502_MOESM2_ESM.pdf]

Supplementary Information for

Species-specific structural adaptation of the potyviral coat protein in virions and virus-like particles

Neža Koritnik, Andreja Kežar, Luka Kavčič, Magda Tušek Žnidarič, Adrijana Leonardi, Swarnalok De, Maija Pollari, Kristiina Mäkinen, Marjetka Podobnik

Correspondence to: kristiina.makinen@helsinki.fi and marjetka.podobnik@ki.si

The PDF file includes:

Supplementary Figures 1 to 28

Supplementary Tables 1 to 3

Supplementary References

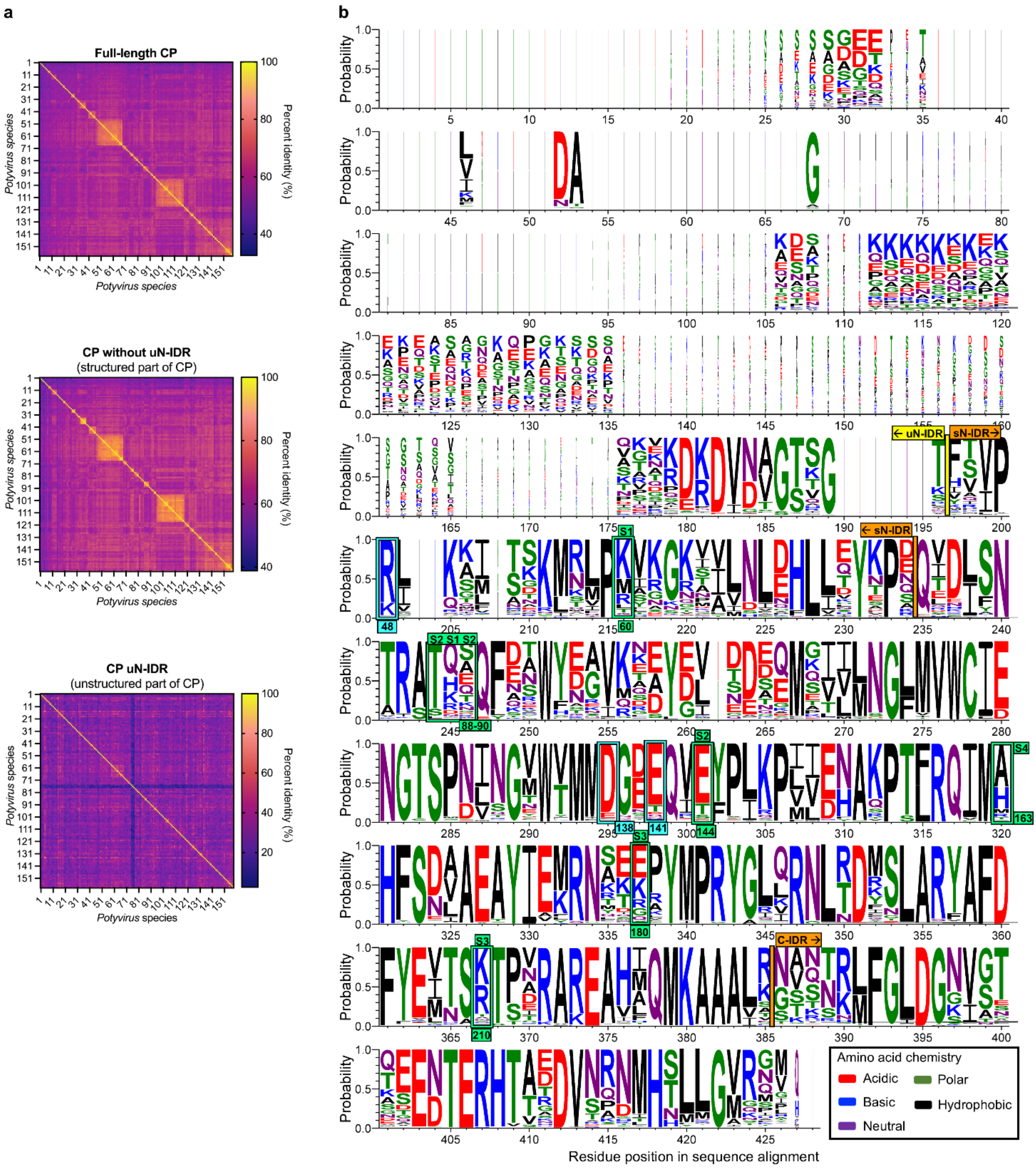

Supplementary Figure 1: Conservation of amino acid sequences in potyviral CPs. a Pairwise identity matrix of 158 potyviral CP sequences. **b** Aligned potyviral CPs (158 complete sequences) presented as sequence logos (created with WebLogo3[1]). Residue heights represent residue probabilities. Green frames highlight the S1-S4 positions in ^{PVA}CP. Cyan frames highlight the positions of the conserved R48, D138, and E141 in ^{PVA}CP. In a and b the CP sequences were taken from phylogenetic tree currently available at (https://ictv.global/system/files/inline-images/OPSR.Pot_.Fig3_.v15.png) - their GenBank accession numbers of used CP sequences are provided in the Supplementary Data 1 file - and aligned using MUSCLE[2].

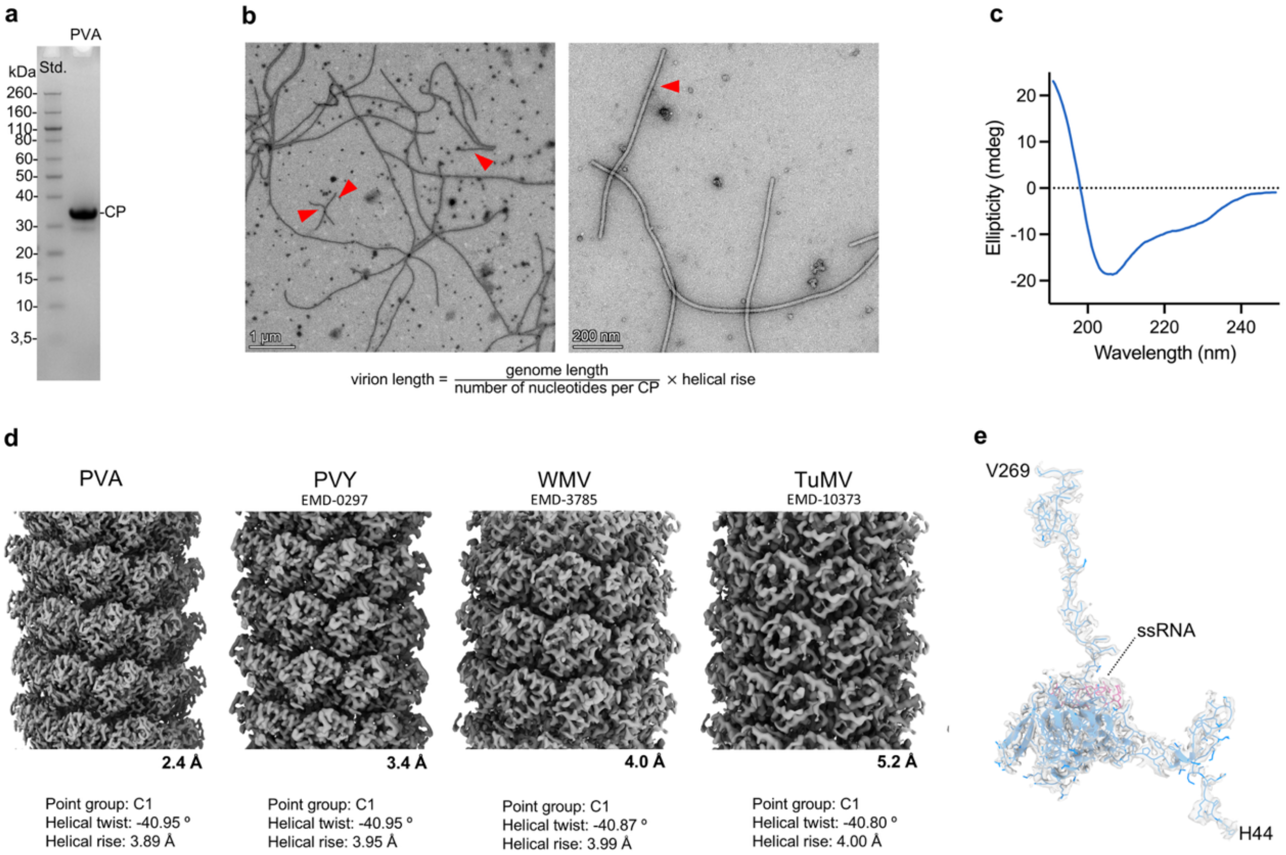

Supplementary Figure 2: Structural characterization of PVA. **a** SDS-PAGE analysis of the purified PVA. **b** Negative stain TEM micrographs of the purified PVA. Red arrows indicate individual particles measuring approximately 840 nm (calculated based on the number of RNA bases in the PVA^{B11} construct carrying Rluc gene with intron[3], see the equation below the micrographs). The longer filaments represent merged PVA particles[4,5]. **c** CD-spectrum of the purified PVA. Data for the curve is provided in the Supplementary Data 1 file. **d** Comparison of cryo-EM density maps of PVA, PVY[6], WMV[7], and TuMV[8], with their overall resolutions and helical symmetry parameters listed below. **e** Fit of the atomic model of a ^{PVA}CP subunit in PVA (blue ribbon and sticks – CP, pink sticks – ssRNA pentanucleotide) into the PVA cryo-EM density map (gray).

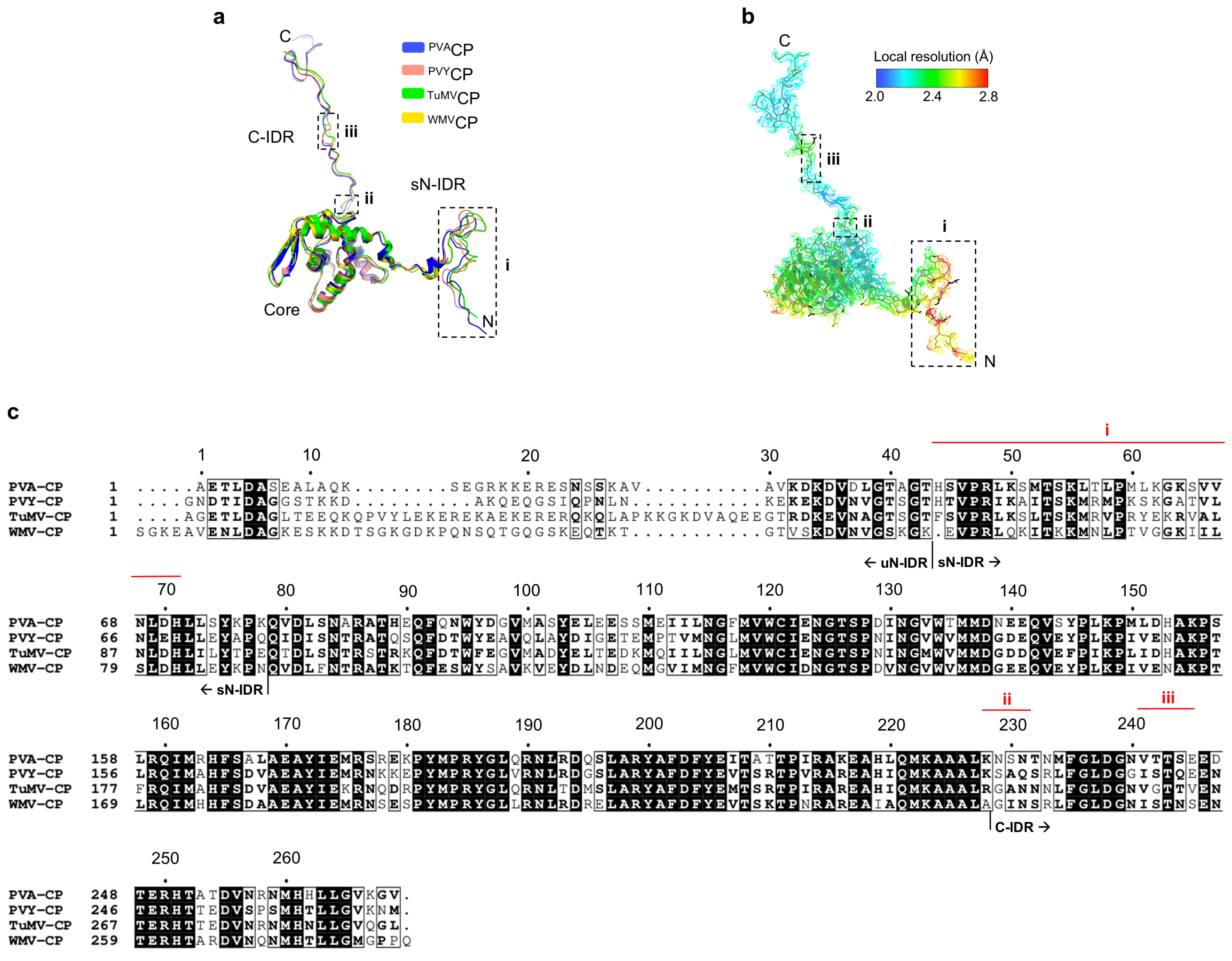

	^{PVA}CP	^{PVY}CP	^{TuMV}CP	^{WMV}CP
^{PVA}CP	**100 %**	59.32 %	63.94 %	58.58 %
^{PVY}CP	59.32 %	**100 %**	62.92 %	64.66 %
^{TuMV}CP	63.94 %	62.92 %	**100 %**	56.16 %
^{WMV}CP	58.58 %	64.66 %	56.16 %	**100 %**

Supplementary Figure 3: Structural and amino acid sequence alignment of CPs from potyviruses with known 3D structures. a Structural superposition on all Cα atoms of $^{\mathrm{PVA\text{-}B11}}$CP, ^{PVY}CP (PDB ID: 6HXX), ^{TuMV}CP (PDB ID: 6T34), and ^{WMV}CP (PDB ID: 5ODV). **b** Fit of the atomic model of a ^{PVA}CP subunit in PVA (black cartoon and sticks) into the PVA cryo-EM density map colored according to the local resolution. In panels a and b, dashed rectangles highlight the regions (i-iii) with low IDR sequence conservation according to the Fig. 1d. **c** Amino acid sequence alignment (conserved residues: black shade; similar residues: bold text; above) and percent identity matrix (below) of potyviral CPs: ^{PVA}CP, ^{PVY}CP, ^{TuMV}CP, ^{WMV}CP. GenBank accession numbers of the aligned CPs are provided in the Supplementary Data 1 file. The alignment was created using the MUSCLE algorithm[2]. Red lines highlight the regions (i-iii) with low IDR sequence conservation according to the Fig. 1d.

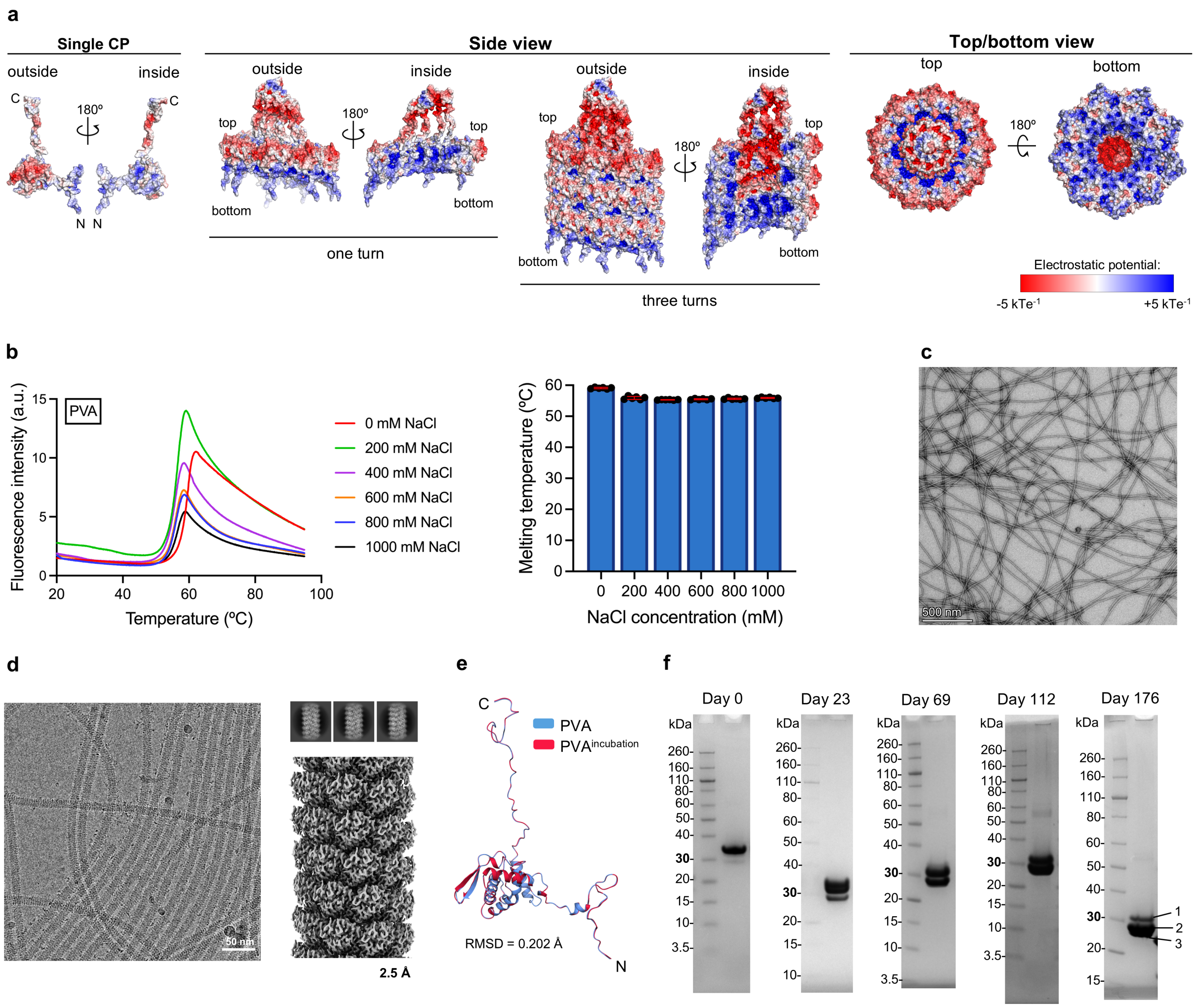

g

Protein band	Edman sequencing	Possible PVACP start region
1	AXTID	AETLD
2	AVAV	AVAV
3	GTASV	GTHSV

PVACP amino acid sequence:

Ala1 Ala28 Gly42

AETLDASEALAQKSEGRKKERESNSSK**AVAV**KDKDVDLGTA**GTHSV**PRLKSMTSKLT
LPMLKGKSVVNLDHLLSYKPKQVDLSNARATHEQFQNWYDGVMASYELEESSMEIIL
NGFMVWCIENGTSPDINGVWTMMDNEEQVSYPLKPMLDHAKPSLRQIMRHFSALAEA
YIEMRSREKPYMPRYGLQRNLRDQSLARYAFDFYEITATTPIRAKEAHLQMKAAALK
NSNTNMFGLDGNVTTSEEDTERHTATDVNRNMHHLLGVKGV

Supplementary Figure 4: Electrostatic surface potential and stability of PVA. a Electrostatic surface potential of ^{PVA}CP in PVA particles (red – negative charge, blue – positive charge), calculated with PDB2PQR[9] with APBS[10]. **b** Thermal stability of PVA at pH 7.0 and different salt concentrations. Left: PVA melting curves (raw data). Melting temperatures (Tm) derived from the melting curves (right). Data are presented as the mean ± standard deviation (red bars) from six measurements (black dots). Data for graphs are provided in the Supplementary Data 1 file. The experiment was done at the same conditions as for PVY thermal stability analysis reported in the Fig 1i of Kavčič *et al*[5]. **c** Negative stain TEM micrograph of PVA after 176 days of incubation at 4 °C (PVA^{incubation}). **d** Cryo-EM analysis of PVA after 176 days of incubation at 4 °C (PVA^{incubation}). Micrograph (left), 2D class averages (top right) and the cryo-EM density map (bottom right) with the overall resolution indicated below in Å. **e** Structural superposition of CPs from PVA and PVA^{incubation} over all Cα atoms. Root mean square (RMSD) value of superposition is shown. **f** SDS-PAGE analysis of PVA at different incubation times at 4 °C. The incubation times are indicated above the gel images. In the final gel (Day 176), three distinct bands (labeled 1, 2, and 3) were identified and analyzed with N-terminal Edman sequencing (panel g). **g** N-terminal Edman sequencing results for bands 1, 2 and 3 from panel f (left), with the corresponding sequence reads mapped to the ^{PVA}CP amino acid sequence (right). 'X' marks the amino acid whose identity could not be experimentally determined.

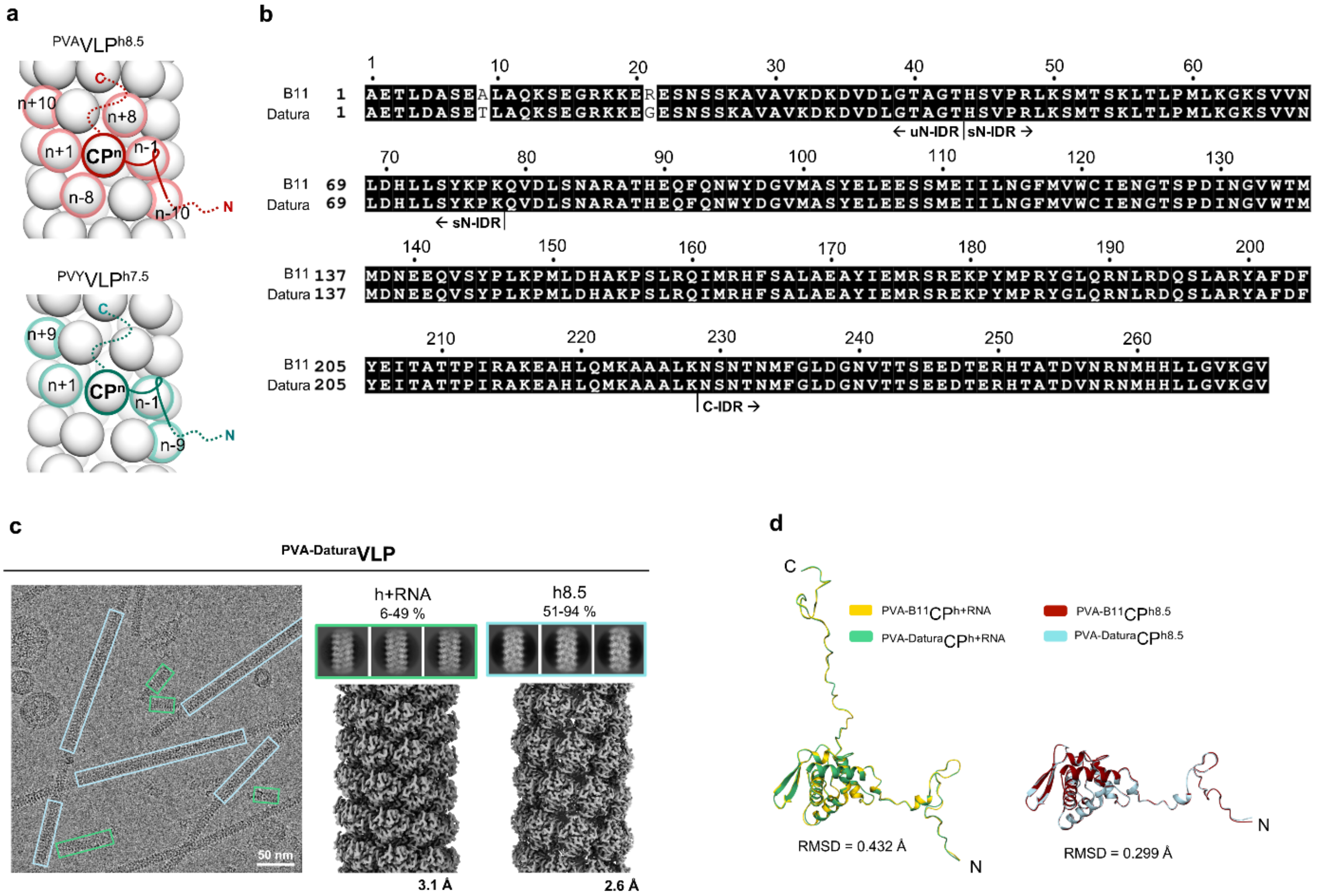

Supplementary Figure 5: CP-CP interactions in ^{PVA}VLP$^{h8.5}$ and ^{PVY}VLP$^{h7.5}$, and comparison of ^{PVA}CPs from PVA isolates B11 and Datura. a Schematic representation of the CP-CP interaction networks in $^{PVA\text{-}B11}$VLP$^{h8.5}$ (above) and ^{PVY}VLP$^{h7.5}$ (below). The focal CP^{n} (dark color) is surrounded by interacting CPs (light color). **b** Amino acid sequence alignment of CPs from PVA^{B11} (GenBank accession number: AJ296311) and PVA^{Datura} (GenBank accession number: Y11426). Identical residues are highlighted in black. The alignment was created using the MUSCLE algorithm[2]. **c** Cryo-EM of $^{PVA\text{-}Datura}$VLPs: cryo-EM micrograph (left), 2D class averages representing the VLP^{h+RNA} and VLP$^{h8.5}$ (top right) with their cryo-EM density maps (bottom right) with their overall resolutions indicated below in Å. Architecturally distinct filaments are highlighted in green (VLP^{h+RNA}) and blue (VLP$^{h8.5}$). **d** Structural superposition (of all Cα atoms) of CP^{h+RNA} (left) and CP$^{h8.5}$s (right) from VLPs formed by CP^{B11} and CP^{Datura}. RMSD values for the aligned Cα atoms are indicated below the superpositions.

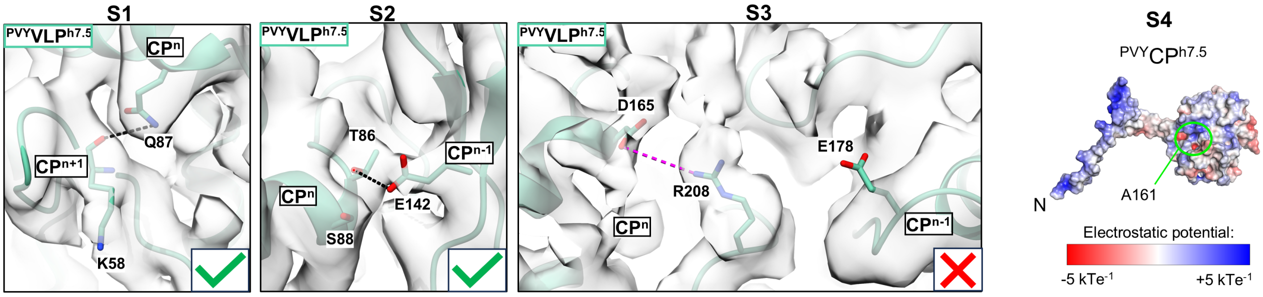

Supplementary Figure 6: Sites S1-S4 in ^{PVY}VLP$^{h7.5}$. Close-up views of CP-CP contacts at sites S1-S3 (left) in the cryo-EM structure of ^{PVY}VLP$^{h7.5}$ (PDB ID: 8OPB), with the cryo-EM density map shown in grey (EMD-17047). The CP model is depicted as a green ribbon, with the labeled amino acid residues shown as sticks. Hydrogen bonds and salt bridges are presented as dotted lines in black and magenta, respectively. Green tick: the CP-CP interaction exists. Red cross: no CP-CP interaction. The site S4 (right) is facing the lumen of ^{PVY}CP$^{h7.5}$ (PDB ID: 8OPB), electrostatic surface potential is shown (red: negative charge, blue: positive charge).

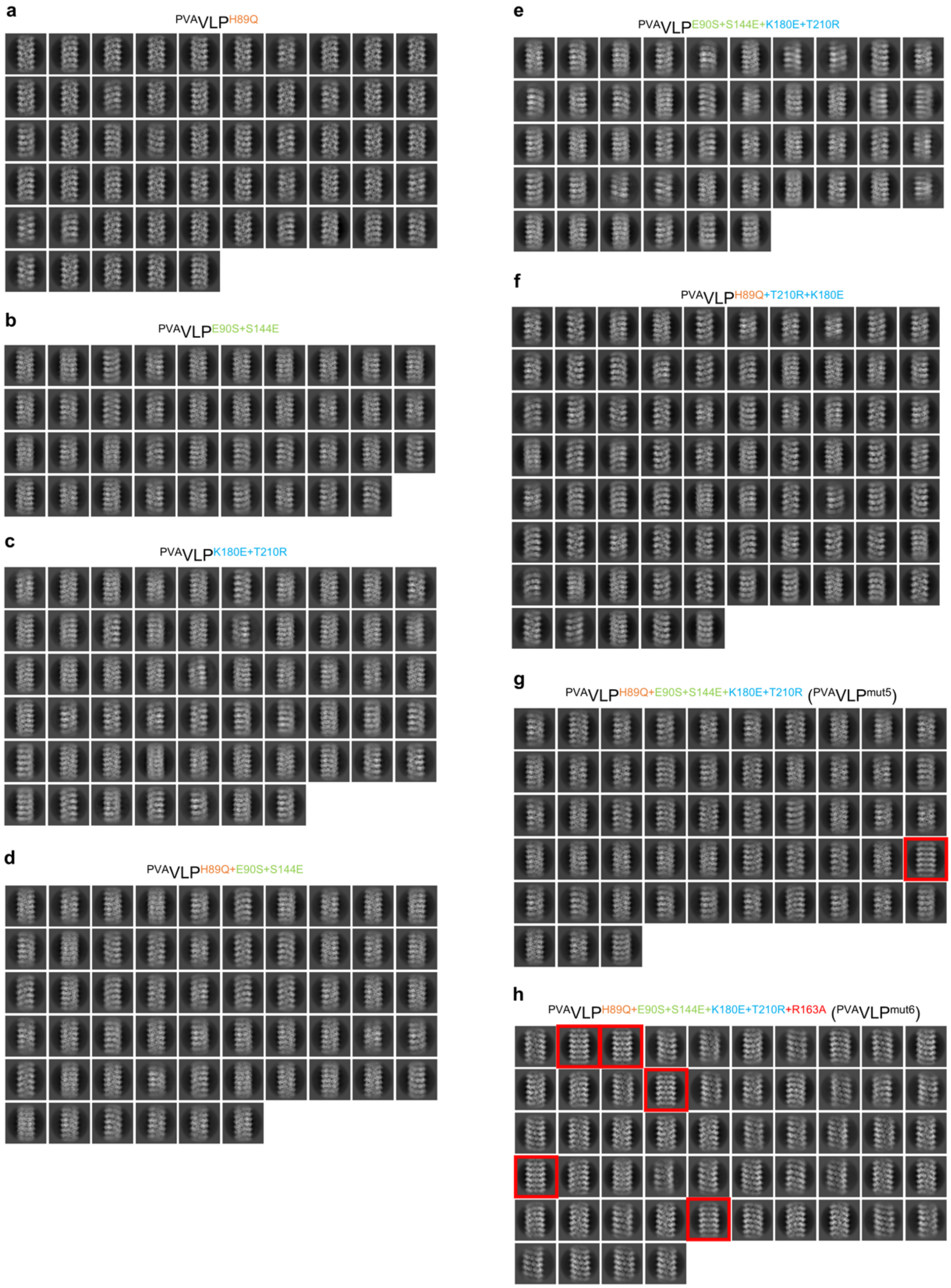

Supplementary Figure 7: 2D class averages of ^{PVA}CP mutants at the sites S1 to S4 and their combinations. Mutations are color-coded: orange – S1, green – S2, blue – S3, and red – S4. In (**a-f**) no stacked-ring VLPs were detected. In (**g, h**) the 2D class averages in red squares mark the stacked-ring VLPs.

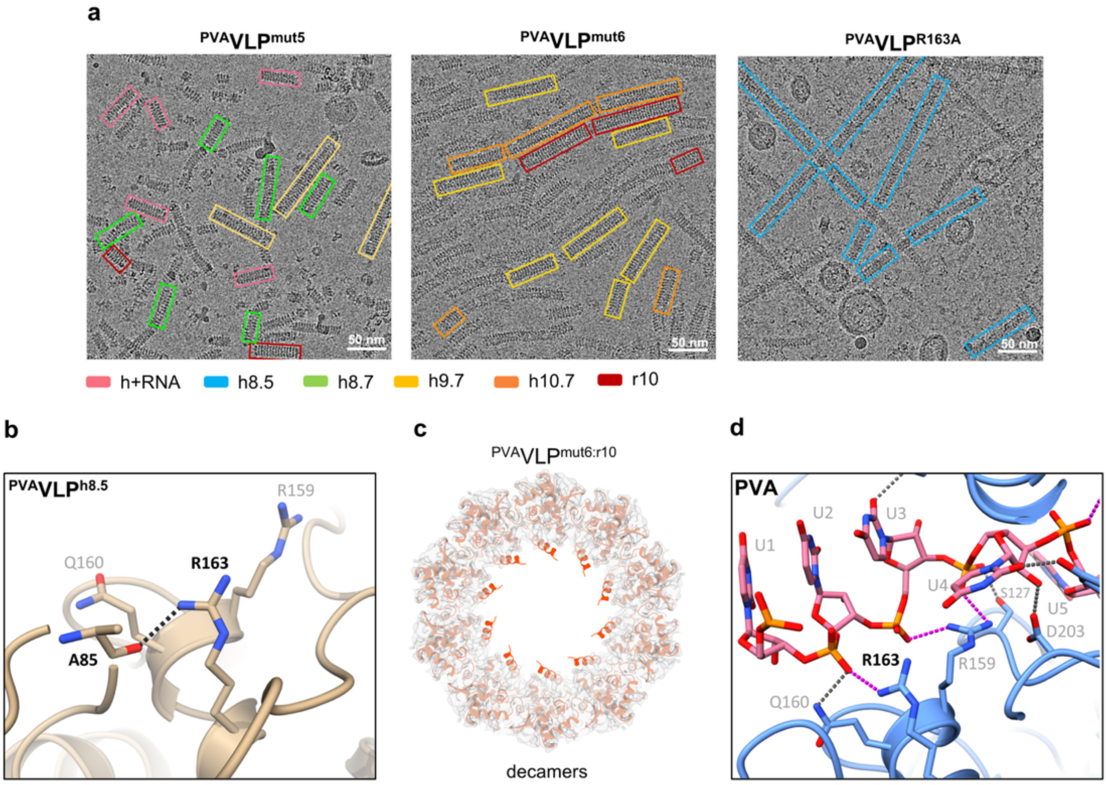

Supplementary Figure 8: Structural analysis of ^{PVA}VLP^{mut5}, ^{PVA}VLP^{mut6}, and ^{PVA}VLP^{R163A}. a Cryo-EM micrographs of ^{PVA}VLP^{mut5}, ^{PVA}VLP^{mut6}, and ^{PVA}VLP^{R163A}. Architecturally distinct filaments are highlighted in pink (VLP^{h+RNA}), light blue (VLP$^{h8.5}$), green (VLP$^{h8.7}$), yellow (VLP$^{h9.7}$), orange (VLP$^{h10.7}$), and dark red (VLP^{r10}). **b** Intramolecular interaction of Arg163 with Ala85 in ^{PVA}VLP$^{h8.5}$. ^{PVA}CP$^{h8.5}$ is depicted in beige. **c** One ring from ^{PVA}VLP$^{mut6:r10}$, depicted as atomic model (orange ribbons), fitted in its cryo-EM density map. **d** RNA-binding pocket of ^{PVA}CP in PVA, with a focus on the interaction between ssRNA and Arg163. ssRNA is depicted in pink and ^{PVA}CP in blue.

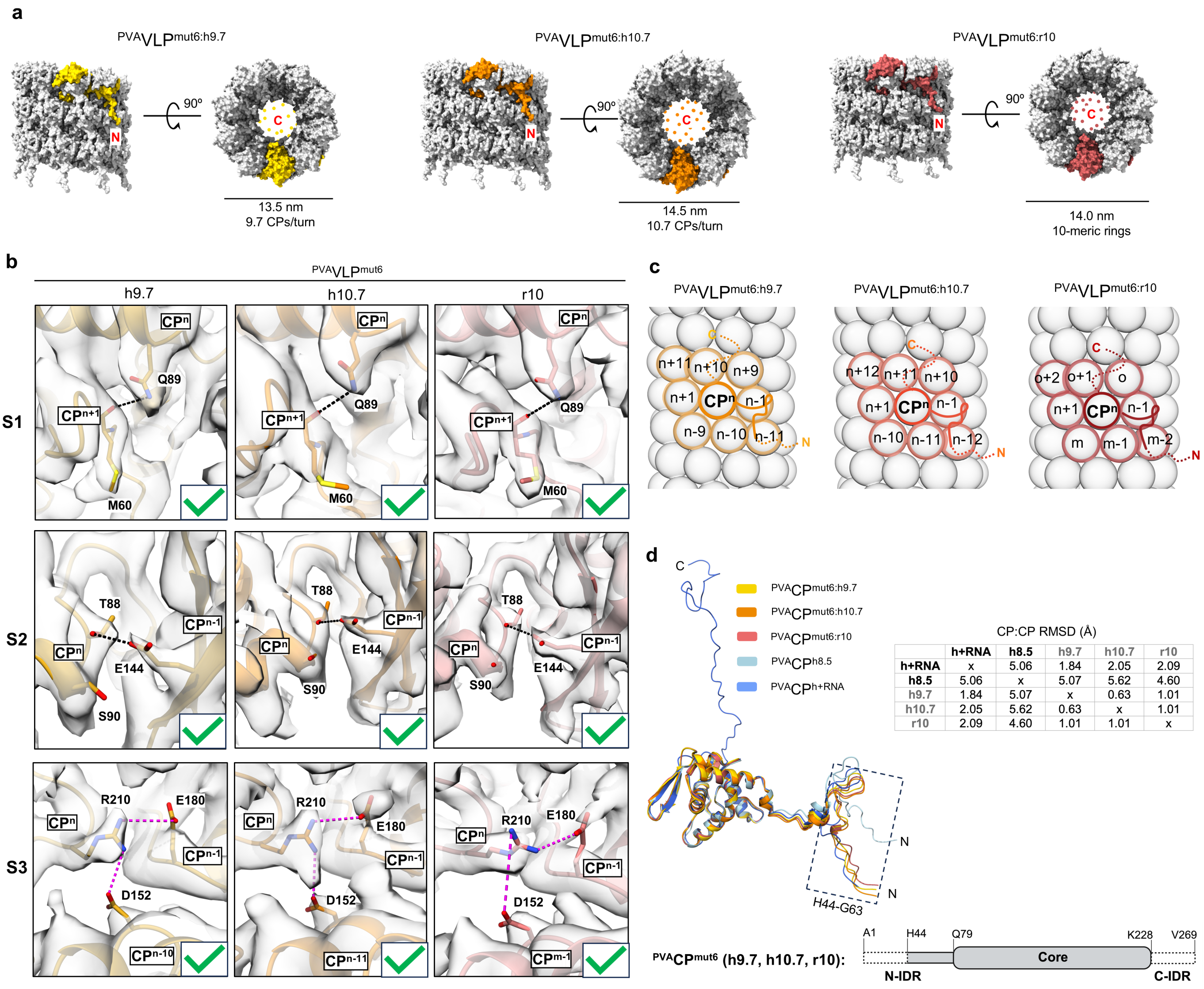

CP:CP RMSD (Å)

	h+RNA	h8.5	h9.7	h10.7	r10
h+RNA	x	5.06	1.84	2.05	2.09
h8.5	5.06	x	5.07	5.62	4.60
h9.7	1.84	5.07	x	0.63	1.01
h10.7	2.05	5.62	0.63	x	1.01
r10	2.09	4.60	1.01	1.01	x

Supplementary Figure 9: Structural analysis of ^{PVA}VLP^{mut6} filaments. **a** Atomic models of ^{PVA}VLP^{mut6} filaments, displayed as surfaces; VLP$^{h9.7}$ (left), VLP$^{h10.7}$(middle), and VLP^{r10} (right). One CP unit is colored yellow (VLP$^{h9.7}$), orange (VLP$^{h10.7}$), and red (VLP^{r10}), other CPs are colored light grey. Dots within the VLP lumens represent the disordered C-IDRs. **b** Close-up views of the sites S1-S3 in structures of ^{PVA}VLP^{mut6} filaments, with atomic models (shown as ribbons) fitted into corresponding cryo-EM density maps. Amino acids of interest are shown as sticks. Hydrogen bonds and salt bridges are presented as dotted lines in black and magenta, respectively. **c** Schematic representation of the CP-CP interaction network in ^{PVA}VLP^{mut6}: VLP$^{h9.7}$ (left), VLP$^{h10.7}$ (center), and VLP^{r10} (right). The focal CP^{n} (dark color) is surrounded by interacting CPs (light color). **d** Structural superposition (on all Cα atoms) of all ^{PVA}VLP^{mut6} CPs (CP$^{h9.7}$, CP$^{h10.7}$ and CP^{r10}) with CP$^{h8.5}$ and CP^{h+RNA} of the wild-type ^{PVA}VLP (above left). Dashed rectangle marks the differences in the sN-IDR kink (His44-Gly63) between the three ^{PVA}CP^{mut6}. RMSD values for the aligned Cα atoms are indicated in the table (above right; names for CP^{mut6} are in grey, and the wild-type CP in black). Schematic representation (created with BioRender.com) of polypeptide chains of all ^{PVA}VLP^{mut6} CPs is shown below in gray. Dashed lines represent the disordered part of the protein structure.

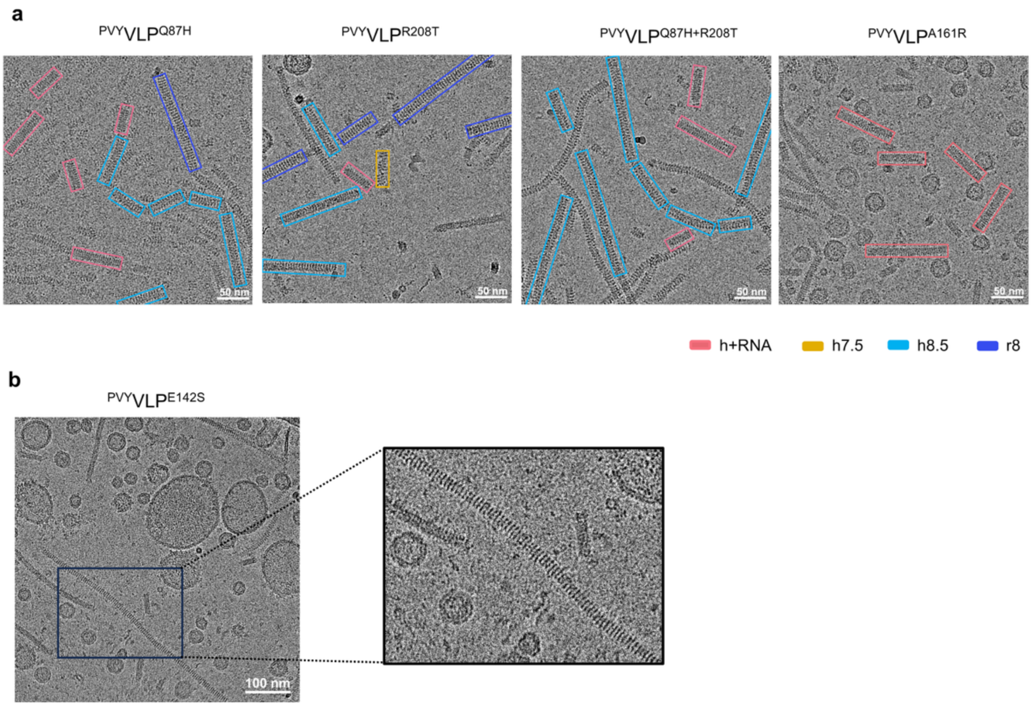

Supplementary Figure 10: Cryo-EM micrographs of ^{PVY}VLP mutants in S1-S4 sites. a Micrographs of ^{PVY}VLP mutants Q87H, R208T, Q87H+R208T, and A161R. Architecturally distinct filaments are highlighted in pink (VLP^{h+RNA}), dark yellow (VLP$^{h7.5}$), light blue (VLP$^{h8.5}$), and dark blue (VLP^{r8}). **b** Cryo-EM micrograph of ^{PVY}VLP^{E142S} (left) with an enlarged view of VLP^{r} (right).

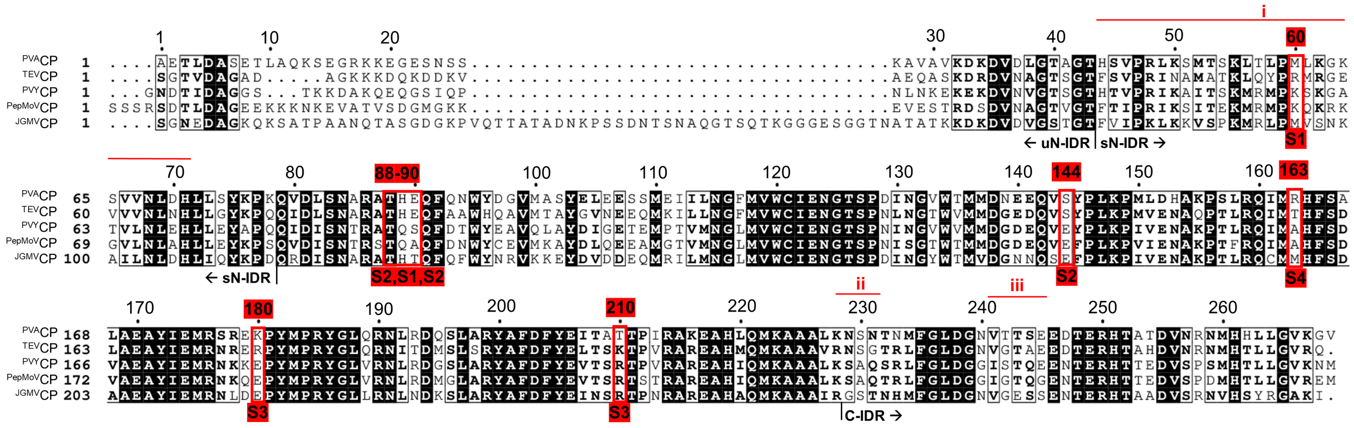

	PVACP	TEVCP	PVYCP	PepMoVCP	JGMVCP
PVACP	**100 %**	65.40 %	59.40 %	56.88 %	57.58 %
TEVCP	65.40 %	**100 %**	61.92 %	58.56 %	56.76 %
PVYCP	59.40 %	61.92 %	**100 %**	74.53 %	57.63 %
PepMoVCP	56.88 %	58.56 %	74.53 %	**100 %**	56.34 %
JGMVCP	57.58 %	56.76 %	57.63 %	56.34 %	**100 %**

Supplementary Figure 11: Comparison of amino acid sequences of CPs from PVA^{B11}, TEV, PVY, PepMoV, and JGMV. Above: Amino acid sequence alignment (GenBank accession numbers provided in the Supplementary Data 1 file). Identical residues are shaded in black. Similar residues are written in bold. The numbering is based on ^{PVA}CP amino acid sequence. Red rectangles highlight amino acid positions in S1-S4. The variable regions i-iii (Fig. 1d) are marked. The alignment was created using the MUSCLE algorithm[2]. Below: Percent identity matrix of sequences from above.

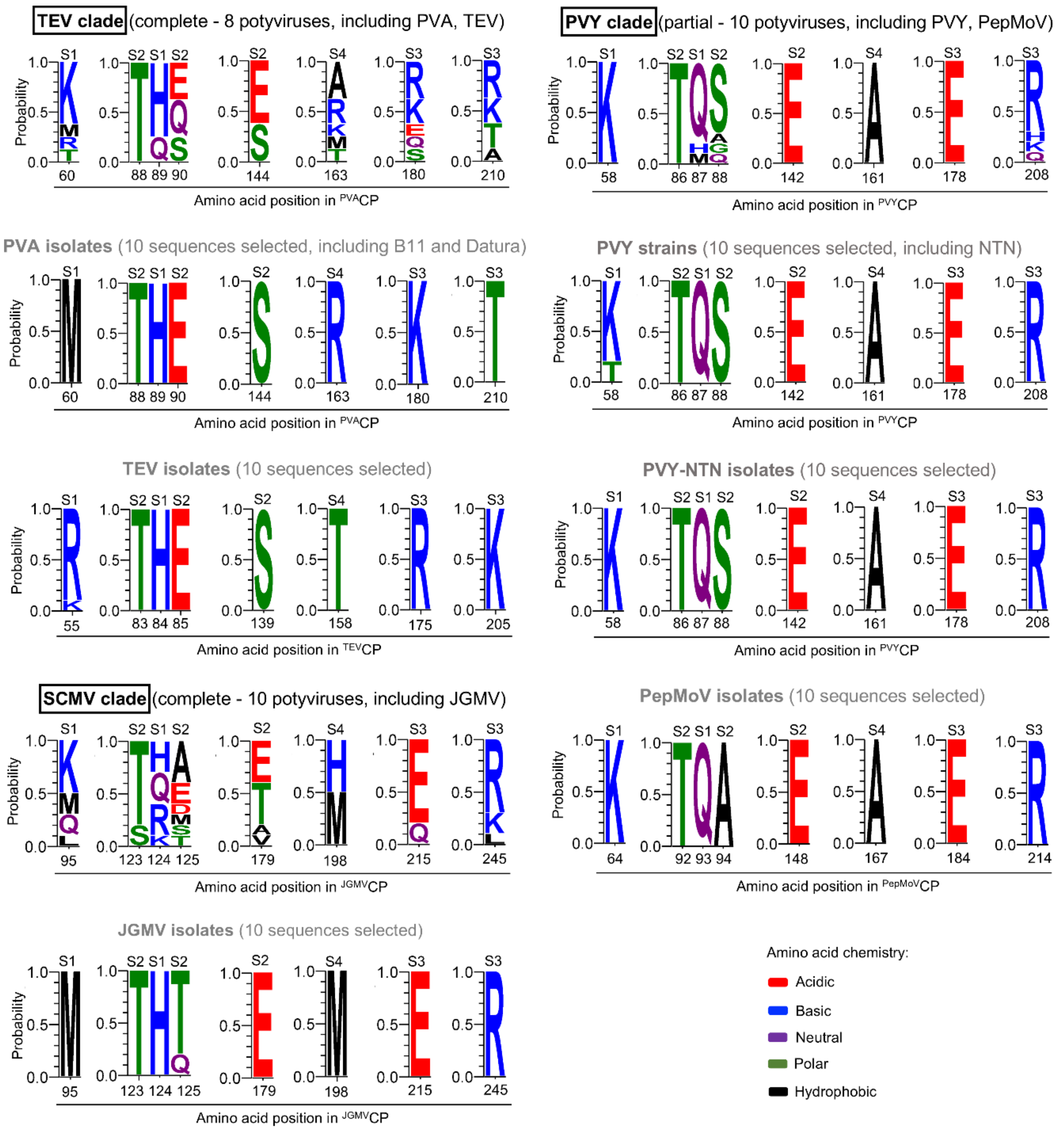

Supplementary Figure 12: Conservation of S1-S4 residues across potyviral phylogenetic clades and isolates/strains. Sequence logos (created using WebLogo 3[1]) illustrating amino acid residue probabilities at positions S1-S4 in potyviral CPs from TEV, PVY, and SCMV (JGMV) clades from phylogenetic tree currently available at (https://ictv.global/system/files/inline-images/OPSR.Pot_.Fig3_.v15.png), as well as from different isolates or strains of PVA, TEV, PVY, PepMoV, and JGMV. For individual clades, ten potyviruses were selected (unless the clade contained less than ten potyviruses). For individual strains/isolates, 10 different sequences were selected from the NCBI Nucleotide database. GenBank accession numbers of used CP sequences are provided in the Supplementary Data 1 file. Residue heights represent residue probabilities.

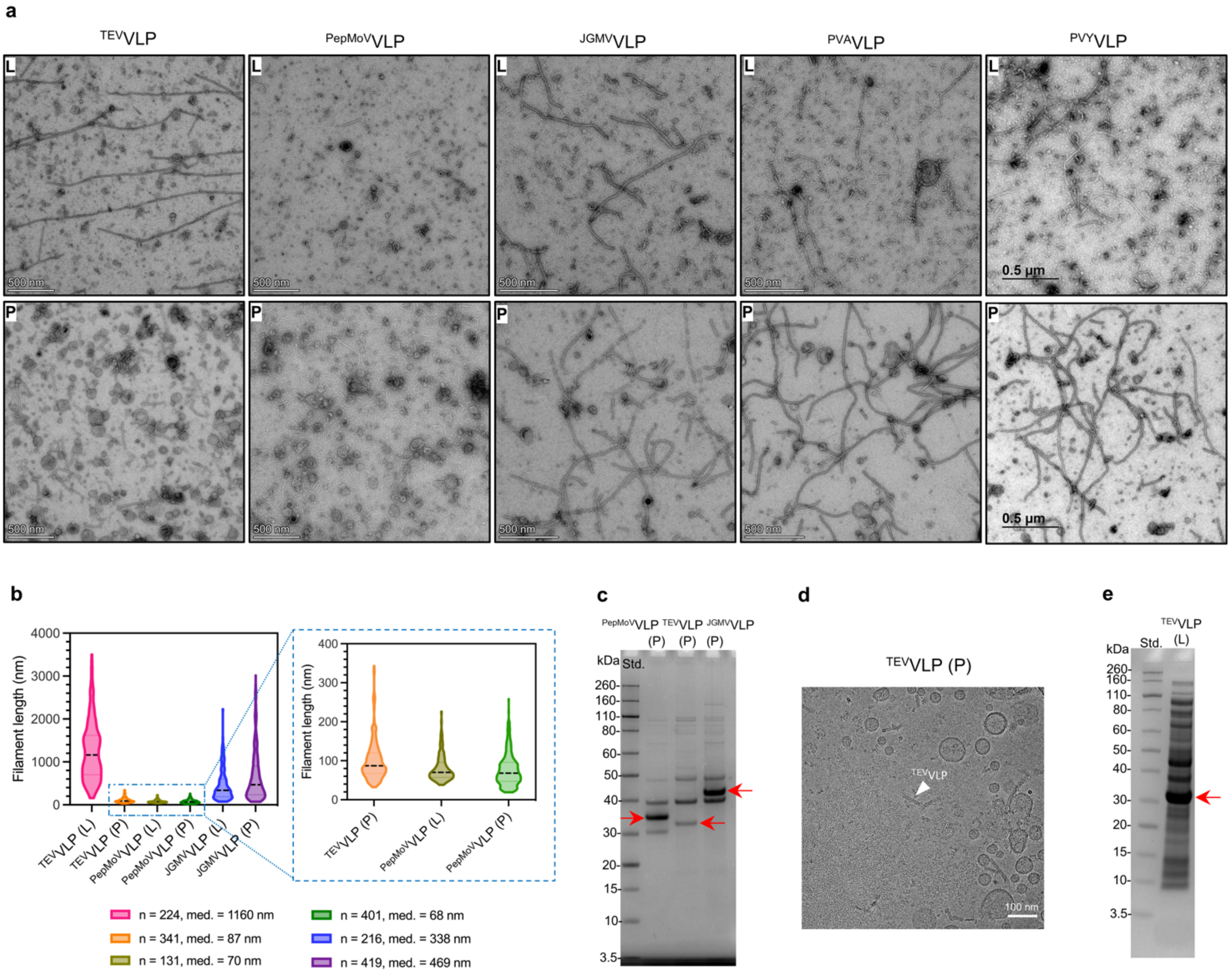

Supplementary Figure 13: Structural characterization of ^{TEV}VLP, ^{PepMoV}VLP, and ^{JGMV}VLP. a nsTEM micrographs of ^{TEV}VLP, ^{PepMoV}VLP, ^{JGMV}VLP, ^{PVA}VLP, and ^{PVY}VLP samples in the soluble fraction of *E. coli* lysate (L) and after purification via precipitation and ultracentrifugation in a sucrose gradient (P). **b** Filament length distribution analysis of ^{TEV}VLPs, ^{PepMoV}VLPs, ^{JGMV}VLPs in L and P samples, presented as violin plots (created with GraphPad Prism v10.2[11]). The number of filaments measured (n) and median filament length (med.) for each distribution are listed in the legend below. Median values are indicated with dashed black lines, while the values of 25th and 75th percentiles are indicated with dashed lines in the same color as their respective plot. Filament measurements are provided in the Supplementary Data 1 file. **c** SDS-PAGE analysis of VLP (P) samples, purified via precipitation and ultracentrifugation in a sucrose gradient. Red arrows mark the CP bands. **d** Cryo-EM micrograph of purified (P) ^{TEV}VLP. **e** SDS-PAGE analysis of sample of ^{TEV}VLP in the *E. coli* lysate soluble fraction (L). Red arrow marks the expected ^{TEV}CP band position.

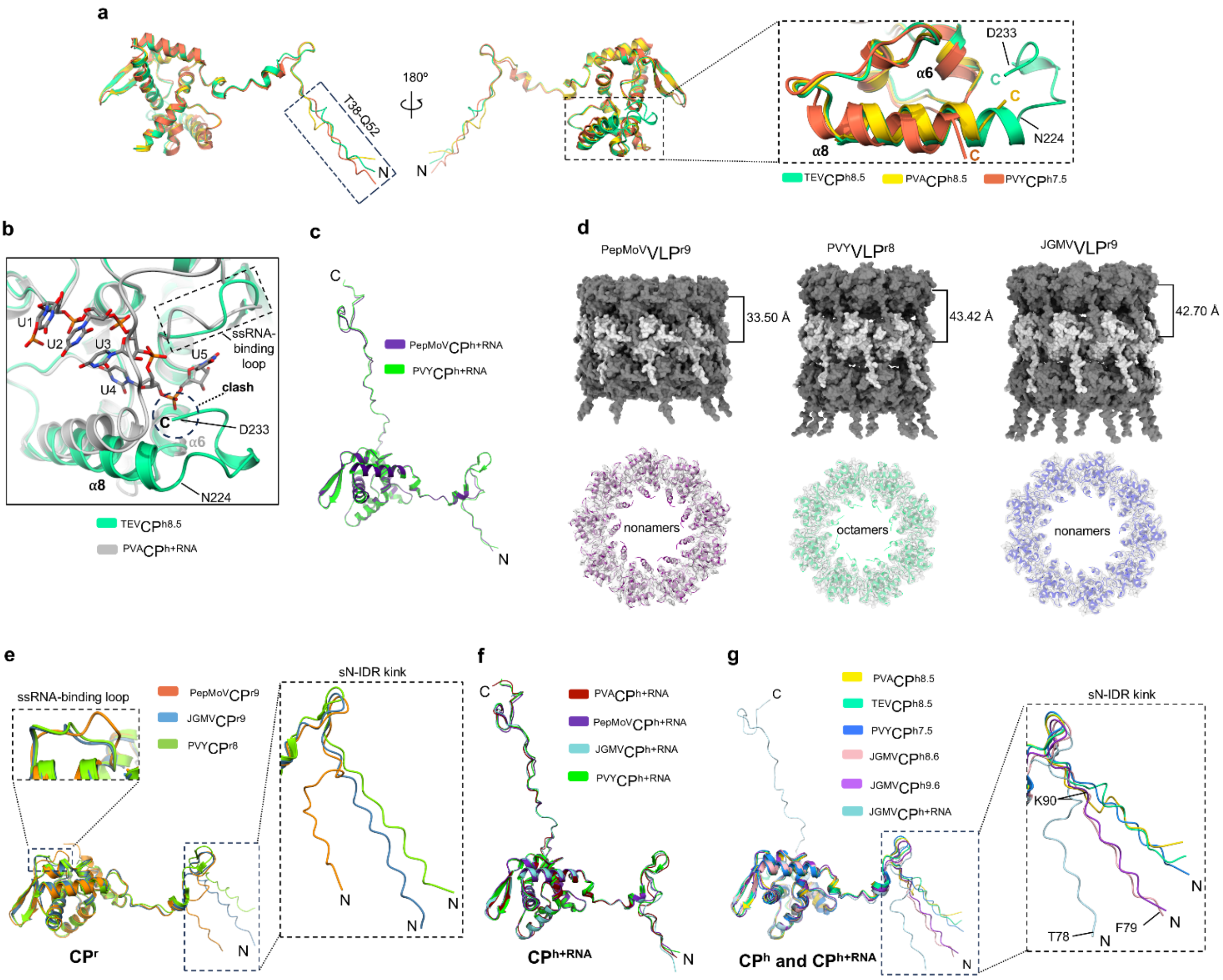

Supplementary Figure 14: Structural characteristics of CPs from TEV, PepMoV, and JGMV VLPs. a Structural superposition (all Cα atoms) of $^{TEV}CP^{h8.5}$, $^{PVA}CP^{h8.5}$, and $^{PVY}CP^{h7.5}$ (PDB ID: 8OPB), with a close-up view of their C-termini. Labeled residues correspond to the $^{TEV}CP^{h8.5}$ structure. **b** A view of the C-termini and ssRNA-binding pockets of superimposed $^{TEV}CP^{h8.5}$ and $^{PVA}CP^{h+RNA}$, depicting how the final structured residues in the $^{TEV}CP^{h8.5}$ prevent RNA binding ('clash'). **c** Structural superposition (on all Cα atoms) of $^{PepMoV}CP^{h+RNA}$ and $^{PVY}CP^{h+RNA}$ (PDB ID: 8OPC). **d** Above: A comparison of helical rises between structures of $^{PepMoV}VLP^{r9}$, $^{JGMV}VLP^{r9}$, and $^{PVY}VLP^{r8}$ (shown as surfaces of their atomic models). Rings are distinguished by different shades of grey. Below: Individual rings from $^{PepMoV}VLP^{r9}$, $^{PVY}VLP^{r8}$ (PDB ID: 8OPA, EMD-17046), and $^{JGMV}VLP^{r9}$, depicted as atomic models fitted in their respective cryo-EM maps. **e** Structural superposition of CPs from different VLP^{r}s: $^{PepMoV}CP^{r9}$, $^{JGMV}CP^{r9}$, and $^{PVY}CP^{r8}$ (PDB ID: 8OPA). **f** Structural superposition of CPs from different VLP^{h+RNA}s: $^{PVA}CP^{h+RNA}$, $^{PepMoV}CP^{h+RNA}$, $^{JGMV}CP^{h+RNA}$, and $^{PVY}CP^{h+RNA}$ (PDB ID: 8OPC). **g** Structural superposition of CPs from different VLP^{h}s: $^{PVA}CP^{h8.5}$, $^{TEV}CP^{h8.5}$, $^{JGMV}CP^{h8.6}$, $^{JGMV}CP^{h9.6}$, and $^{PVY}CP^{h7.5}$ (PDB ID: 8OPB) with $^{JGMV}CP^{h+RNA}$. Labeled residues correspond to the $^{JGMV}CP^{h8.6}$, $^{JGMV}CP^{h9.6}$, and $^{JGMV}CP^{h+RNA}$ structures.

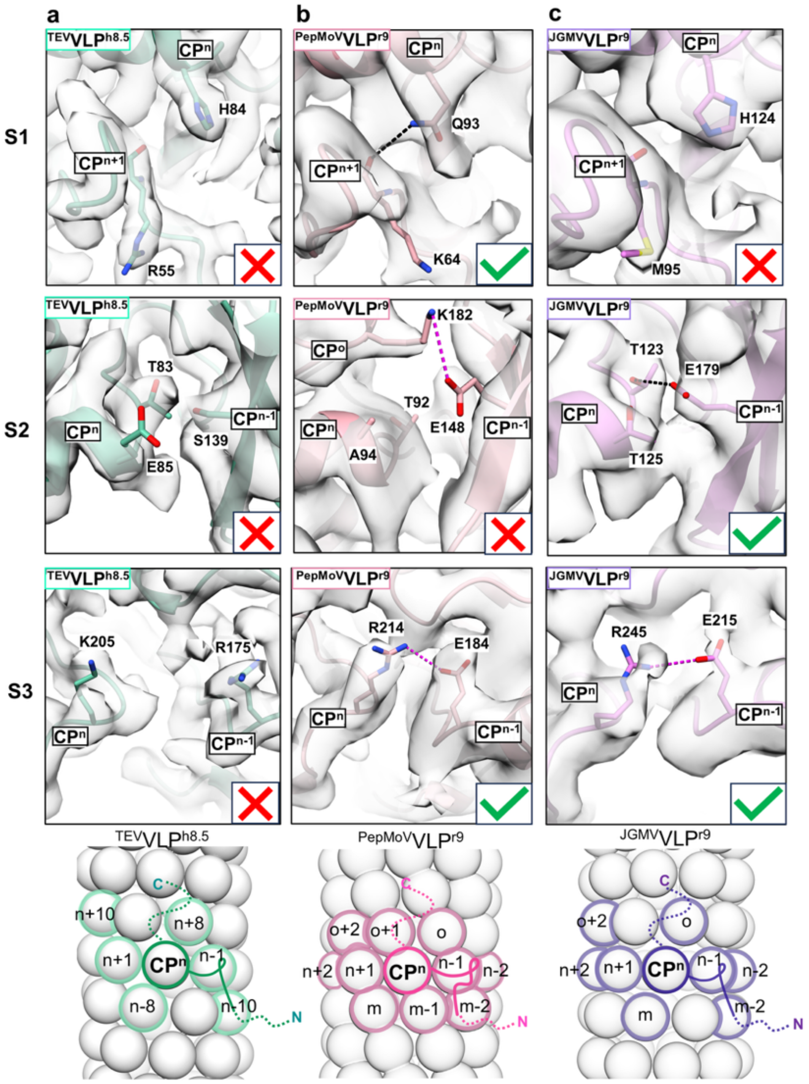

Supplementary Figure 15: Visualization of the sites S1-S3 in the cryo-EM structures of ^{TEV}VLP$^{h8.5}$, ^{PepMoV}VLP^{r9} and ^{JGMV}VLP^{r9}. a-c Close-up views of S1-S3 in structural models (ribbons) of ^{TEV}VLP$^{h8.5}$ (a), ^{PepMoV}VLP^{r9} (b), and ^{JGMV}VLP^{r9} (c) fitted into their corresponding cryo-EM density maps. The amino acid residues of interest are shown as sticks. Hydrogen bonds and salt bridges are presented as dotted lines in black and magenta, respectively. Red crosses: no interaction; green tick: interaction exists. Bellow: schematic representation of the CP-CP interaction networks in ^{TEV}VLP$^{h8.5}$ (a), ^{PepMoV}VLP^{r9} (b), and ^{JGMV}VLP^{r9} (c). The focal CP^{n} (dark color) is surrounded by interacting CPs (light color).

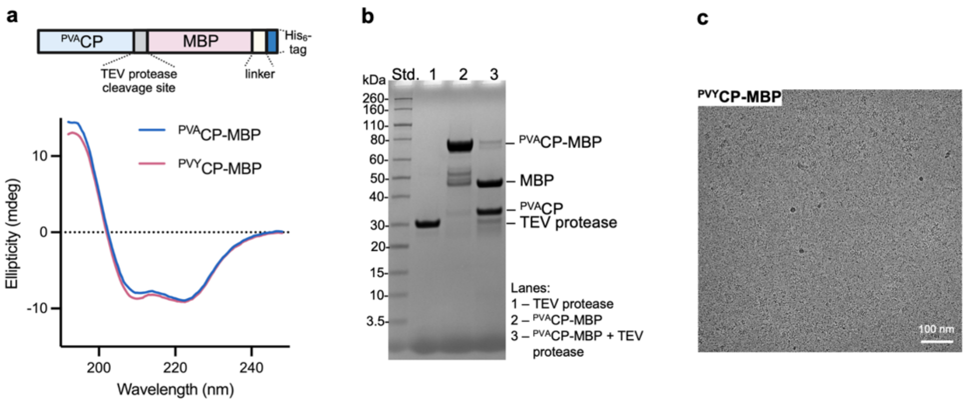

Supplementary Figure 16: Preparation of CP-MBP. a Above: Schematic representation (created with BioRender.com) of ^{PVA}CP-MBP polypeptide chain. Below: CD-spectrum of ^{PVA}CP-MBP and ^{PVY}CP-MBP (curve data provided in the Supplementary Data 1 file). **b** SDS-PAGE analysis of ^{PVA}CP-MBP before and after cleavage by TEV protease. **c** Cryo-EM micrograph of ^{PVY}CP-MBP.

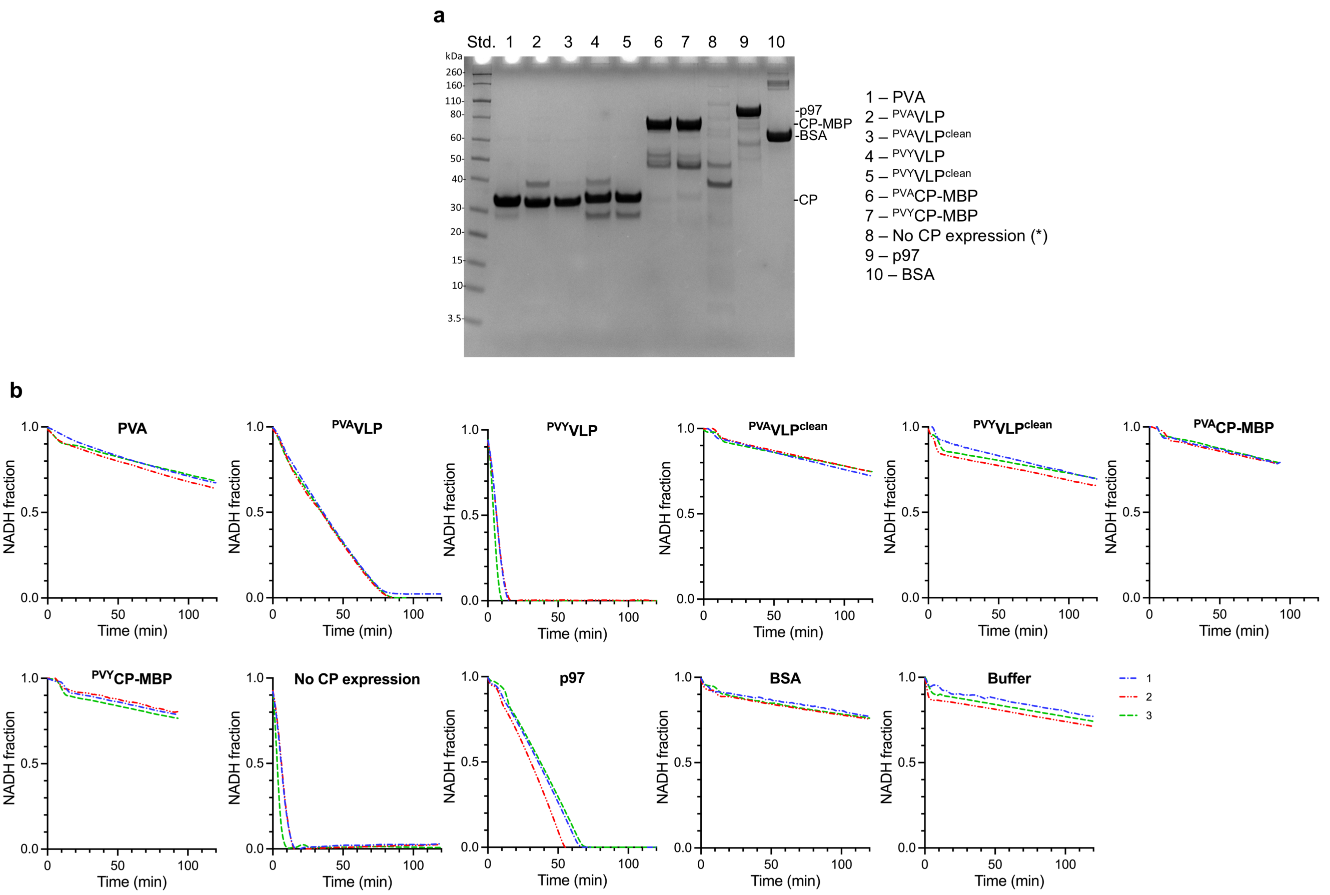

Supplementary Figure 17. Additional data for ATPase assay. a SDS-PAGE analysis of all proteins used in ATPase assays. (*): soluble *E. coli* lysate in absence of CP expression after VLP purification procedure (precipitation and ultracentrifugation in a sucrose gradient). **b** Three experimental repeats (1, 2, 3) of ATPase assays. ATP hydrolysis is presented as NADH depletion over time[12]. Data for curves are provided in the Supplementary Data 1 file.

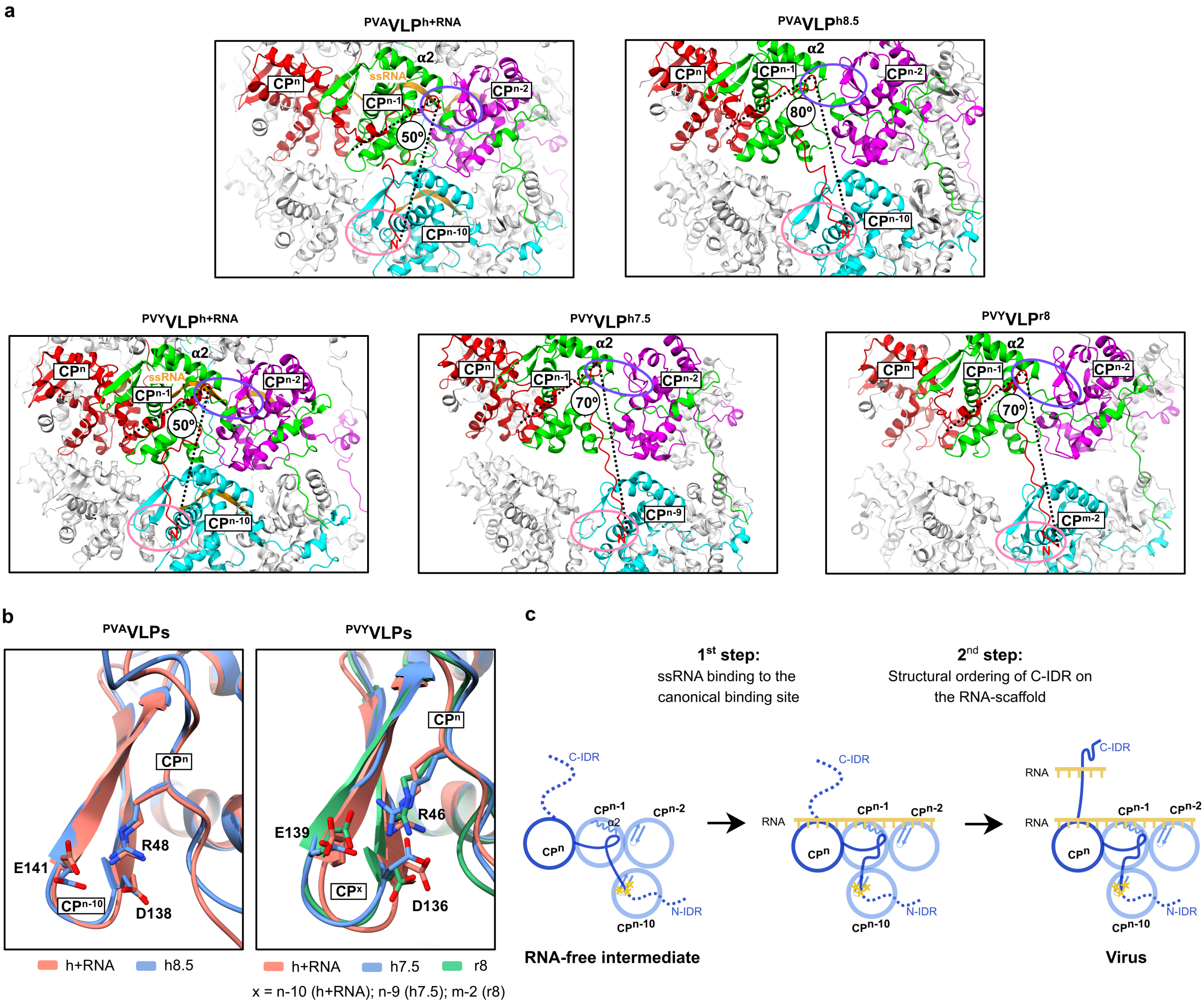

Supplementary Figure 18: Structural role of the highly conserved charged triad and a model of species-specific assembly of virions. a Visualization of the sN-IDR kink angles and interactions with the neighboring CPs within the atomic structures of ^{PVA}VLPs (or VLP^{h+RNA}) and VLP$^{h8.5}$; above) and ^{PVY}VLPs (or VLP^{h+RNA} (PDB ID: 8OPC)), VLP$^{h7.5}$ (PDB ID: 8OPB), and VLP^{r8} (PDB ID: 8OPA); below). sN-IDR kink region has different conformations in the presence or absence of ssRNA binding. However, in both cases, the sN-IDR of CP^{n} is locked in the respective position with the interaction between the conserved Arg48 (^{PVA}CP) and the conserved Asp138 and Glu141 (^{PVA}CP) from the β-hairpin of CP^{n-10} (depicted by the pink ellipse). In addition, the sN-IDR kink is also indirectly associated with the β-hairpin of CP^{n-2} (via α2 helix of CP^{n-1}) (purple ellipse). **b** Close-up view of the conserved Arg-Asp-Glu triad in the atomic structures of PVA/^{PVA}VLP^{h+RNA}s and VLP$^{h8.5}$ (left) and PVY/^{PVY}VLP^{h+RNA} (PDB ID: 8OPC), ^{PVY}VLP$^{h7.5}$ (PDB ID: 8OPB), and ^{PVY}VLP^{r8} (PDB ID: 8OPA); right). **c** Schematic model of the proposed species-specific assembly of virions/VLPs: in the late stages of infections (at high CP concentrations), CPs start assembling into ordered oligomers with structural parameters similar to those found in RNA-free VLPs. The highly conserved charged triad (yellow stars) enables the stability of RNA-free assemblies (as shown for ^{PVA}VLPs (Fig. 6a) and ^{PVY}VLPs[5]). The homogeneity (in terms of CP identity) and stability of RNA-free assemblies is achieved through species-specific CP-CP interactions influenced by the S1-S4 sites. Binding of ssRNA into the canonical ssRNA-binding site triggers the movement of adjacent CP^{n}, CP^{n-2} and CP^{n-10} units, and the shift of the sN-IDR kink in CP^{n} from the open (RNA-free) to the closed '(RNA-bound) conformation, along with the reorganization of C-IDR and its binding to ssRNA at the non-canonical ssRNA binding site, leading to the formation of virions.

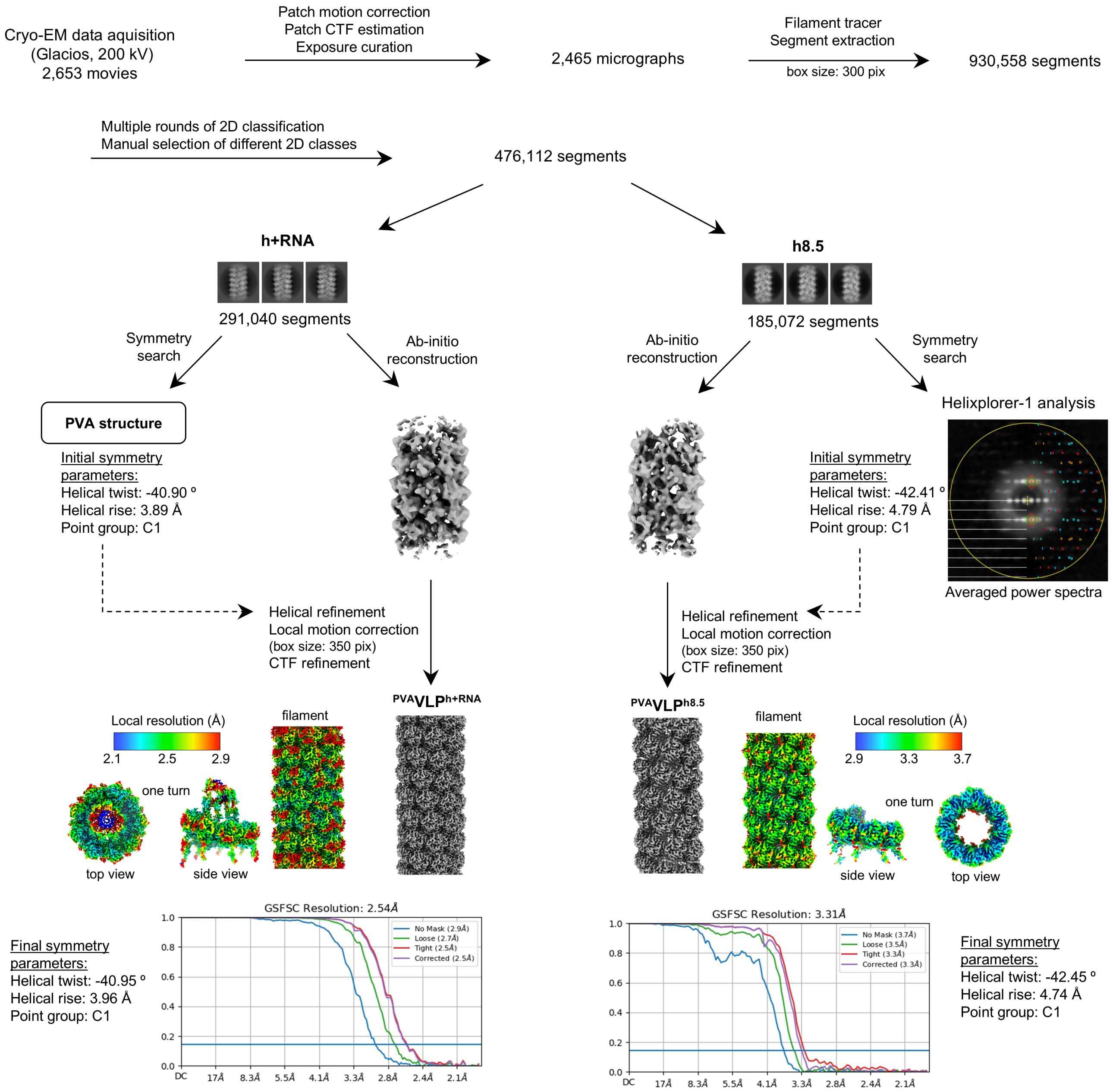

Supplementary Figure 19: Schematic presentation of cryo-EM data processing of PVA-B11VLPs in CryoSPARC v4.3. Initial helical symmetry parameters for PVA structure reconstruction were obtained from PVY structure (EMD-0297, Supplementary Data 2. Cryo-EM data processing for all other datasets in this study followed the same protocol. Numeric data from each workflow are summarized in Supplementary Data 2. FSC curves and local resolution maps for other structures (not presented in this scheme or in Supplementary Fig. 20) are provided in Supplementary Fig. 21 with corresponding Helixplorer-1 analyses shown in Supplementary Fig. 22.

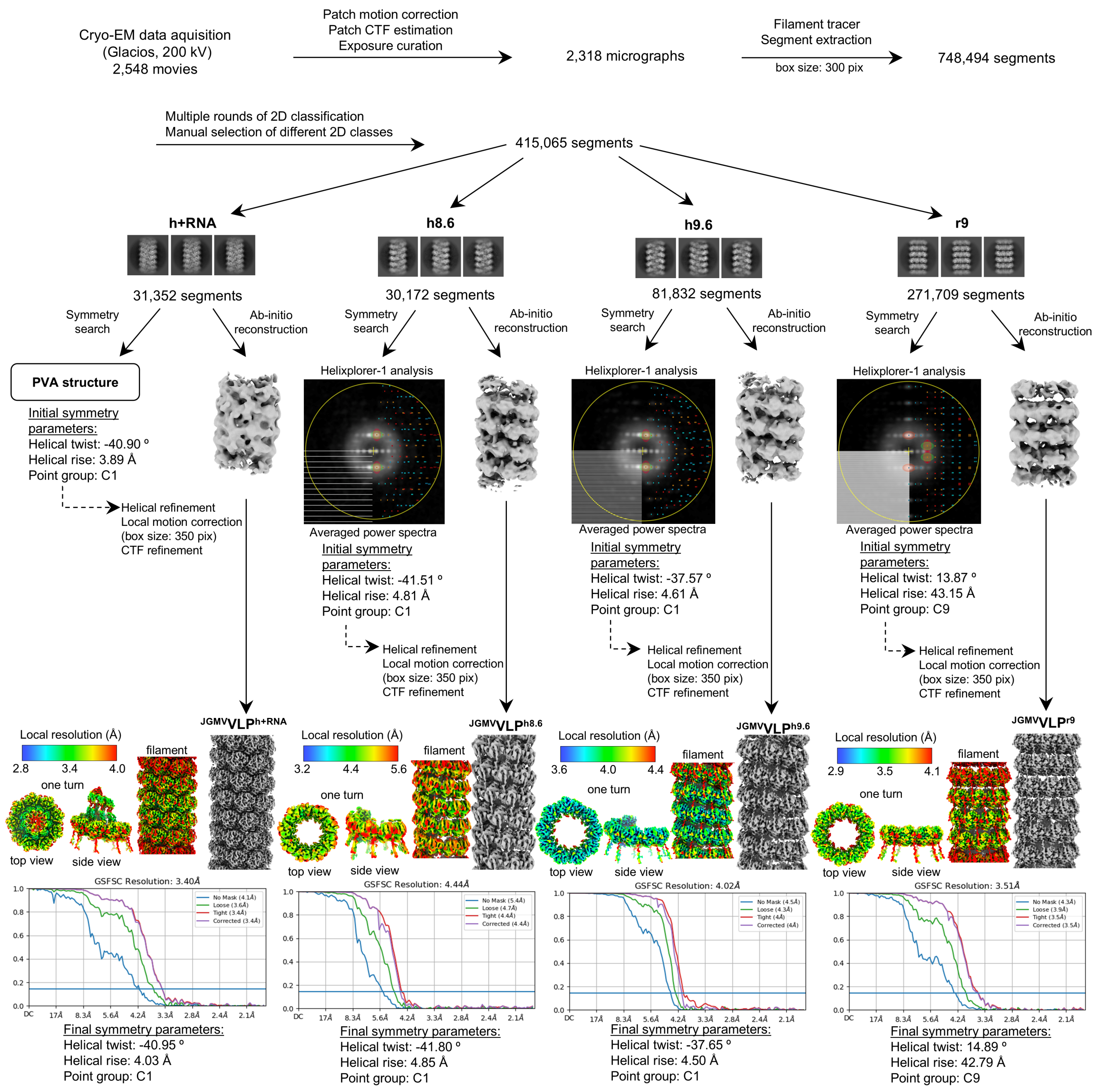

Supplementary Figure 20: Schematic presentation of cryo-EM data processing of ^{JGMV}VLPs in CryoSPARC v4.3. Initial helical symmetry parameters for PVA structure reconstruction were obtained from PVY structure (EMD-0297, Supplementary Data 2. Cryo-EM data processing for all other datasets in this study followed the same protocol. Numeric data from each workflow are summarized in Supplementary Data 2. FSC curves and local resolution maps for other structures (not presented in this scheme or in Supplementary Fig. 19) are provided in Supplementary Fig. 21, with corresponding Helixplorer-1 analyses in Supplementary Fig. 22.

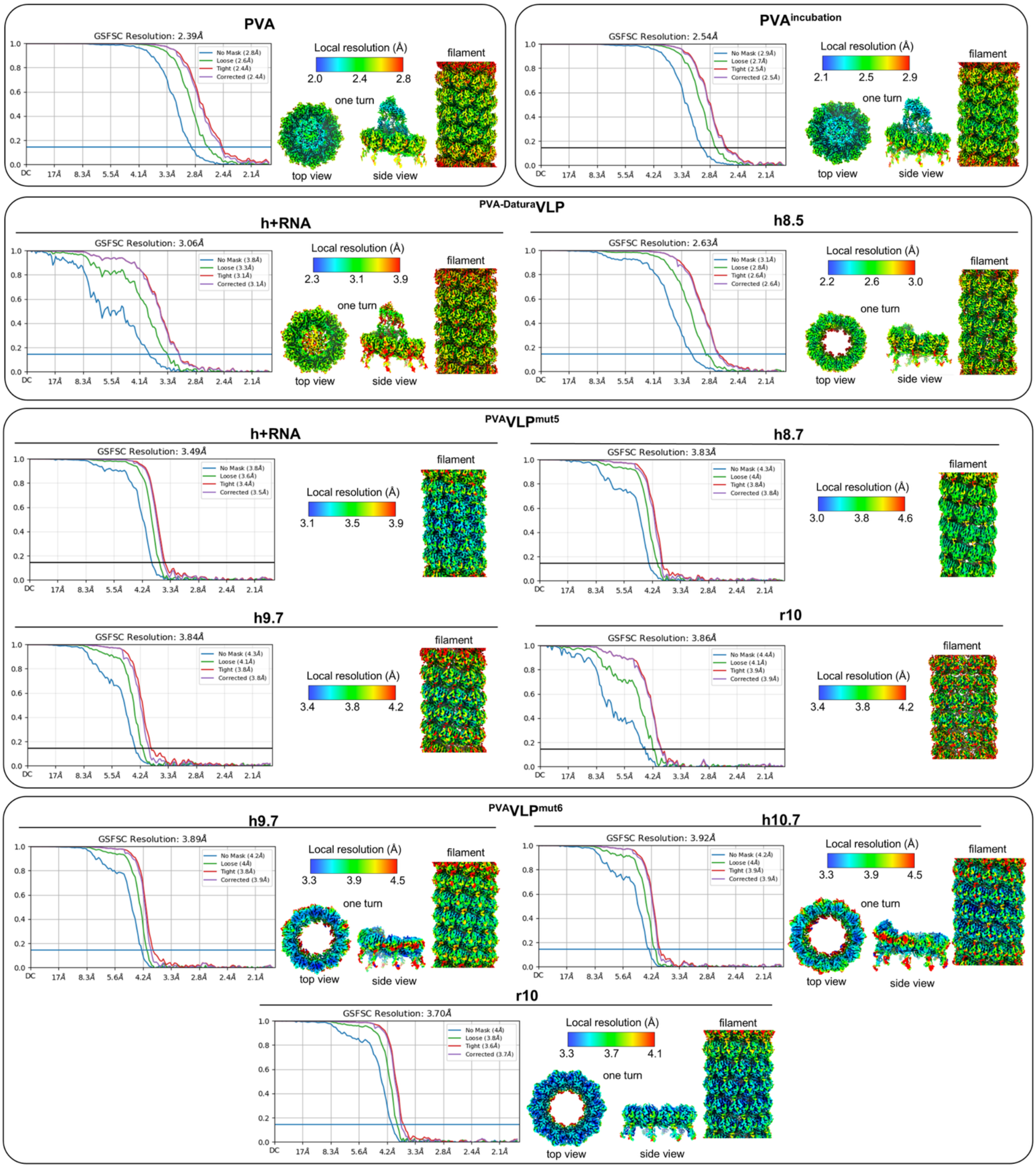

This figure (Supplementary Figure 21) is continued on the next page.

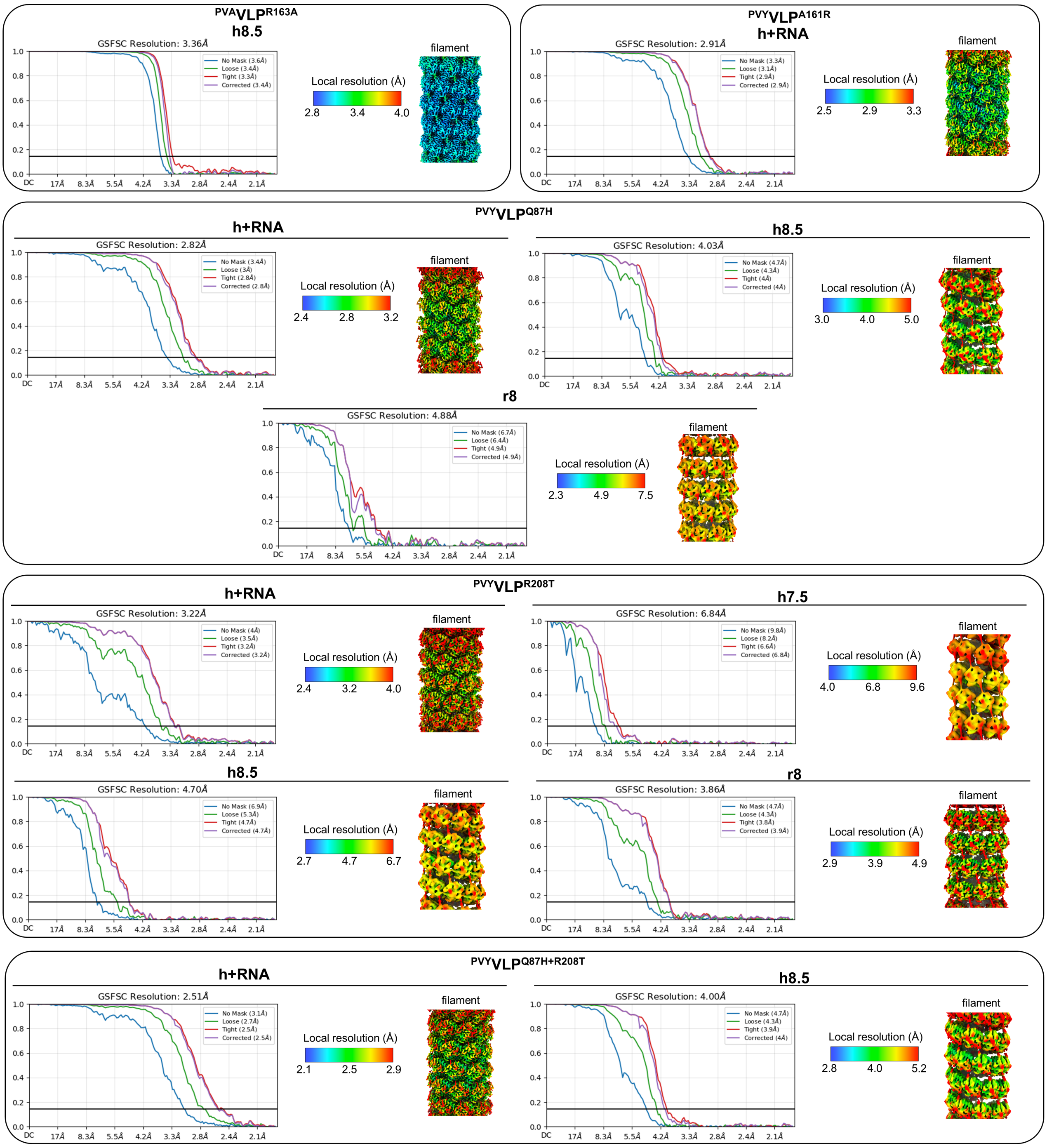

This figure (Supplementary Figure 21) is continued on the next page.

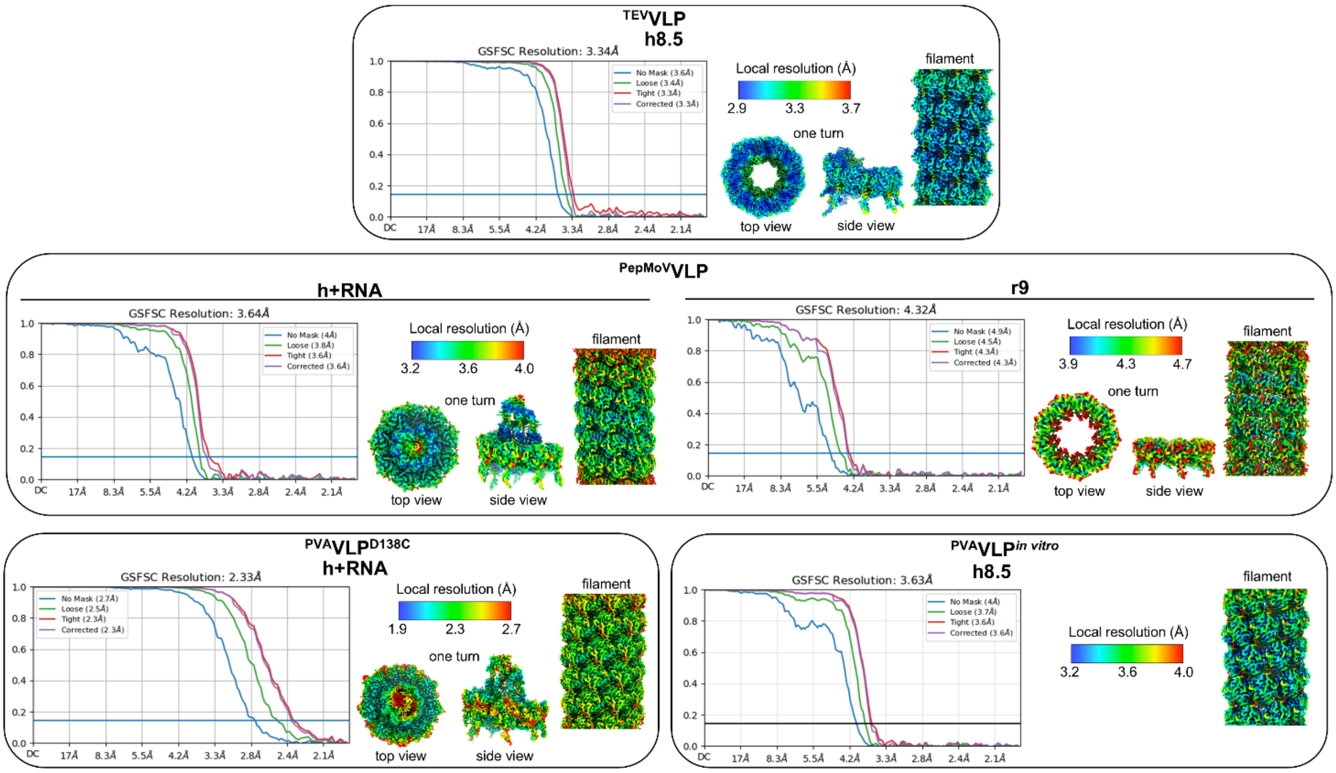

Supplementary Figure 21: Fourier shell correlation (FSC) curves and local resolution maps of the structures of PVA, PVA^{incubation}, $^{\text{PVA-Datura}}$VLPs, ^{PVA}VLP$^{\text{mut5}}$s, ^{PVA}VLP$^{\text{mut6}}$s, ^{PVA}VLP$^{\text{R163A}}$, ^{PVY}VLP$^{\text{A161R}}$, ^{PVY}VLP$^{\text{Q87H}}$s, ^{PVY}VLP$^{\text{R208T}}$s, ^{PVY}VLP$^{\text{Q87H+R208T}}$s, ^{TEV}VLP, ^{PepMoV}VLPs, ^{PVA}VLP$^{\text{D138C}}$, and ^{PVA}VLP$^{\textit{in vitro}}$. The FSC curves (left) show the overall resolution of the structures. Cryo-EM density maps (right) are colored according to their local resolution. Local resolution maps are presented as one helical turn (in case of a resolved atomic model) and as filament.

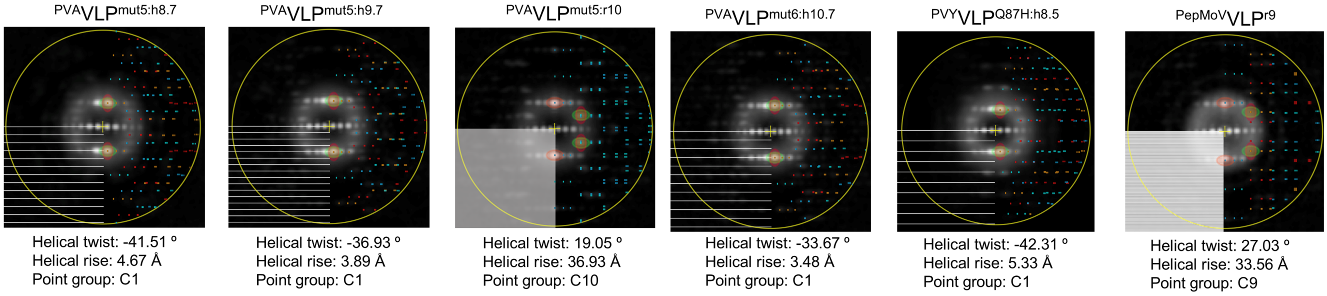

Supplementary Figure 22: Estimation of initial helical symmetry parameters from the averaged power spectra of the structures of $^{PVA}VLP^{mut5:h8.7}$, $^{PVA}VLP^{mut5:h9.7}$, $^{PVA}VLP^{mut5:r10}$, $^{PVA}VLP^{mut6:h10.7}$, $^{PVY}VLP^{Q87H:h8.5}$, and $^{PepMoV}VLP^{r9}$. Helixplorer-1[13] was used to analyze the averaged power spectra, providing initial estimates of the helical symmetry parameters listed below the spectra and in Supplementary Data 2.

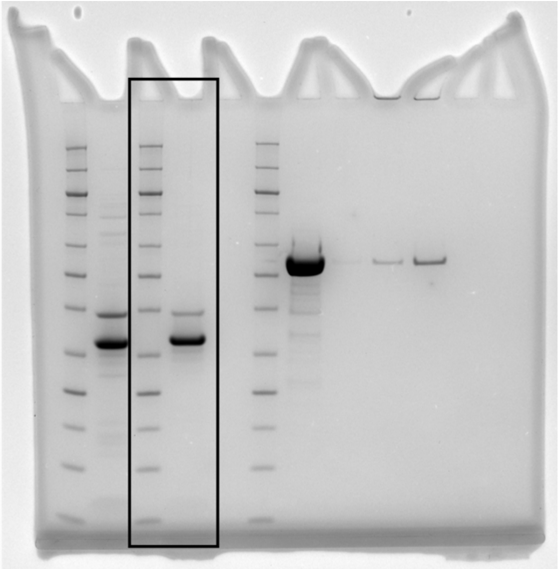

Supplementary Figure 23: Uncropped SDS-PAGE gel of the ^{PVA}VLP sample, related to Fig. 6e. The black frame marks the part of the gel shown in Fig. 6e.

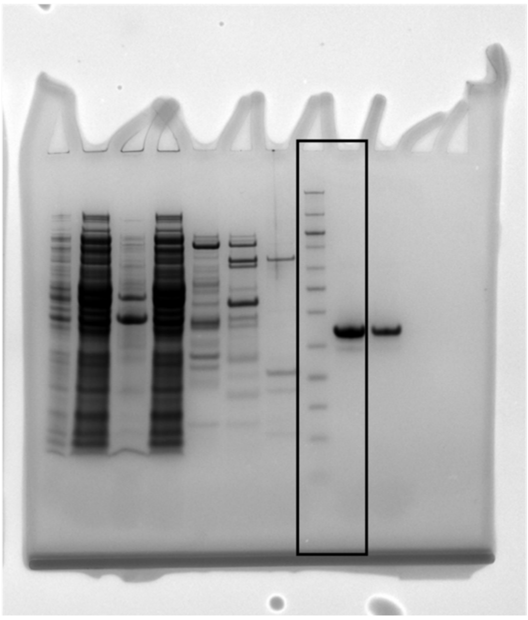

Supplementary Figure 24: Uncropped SDS-PAGE gel of purified PVA, related to Supplementary Fig. 2a. The black frame marks the part of the gel shown in Supplementary Fig. 2a.

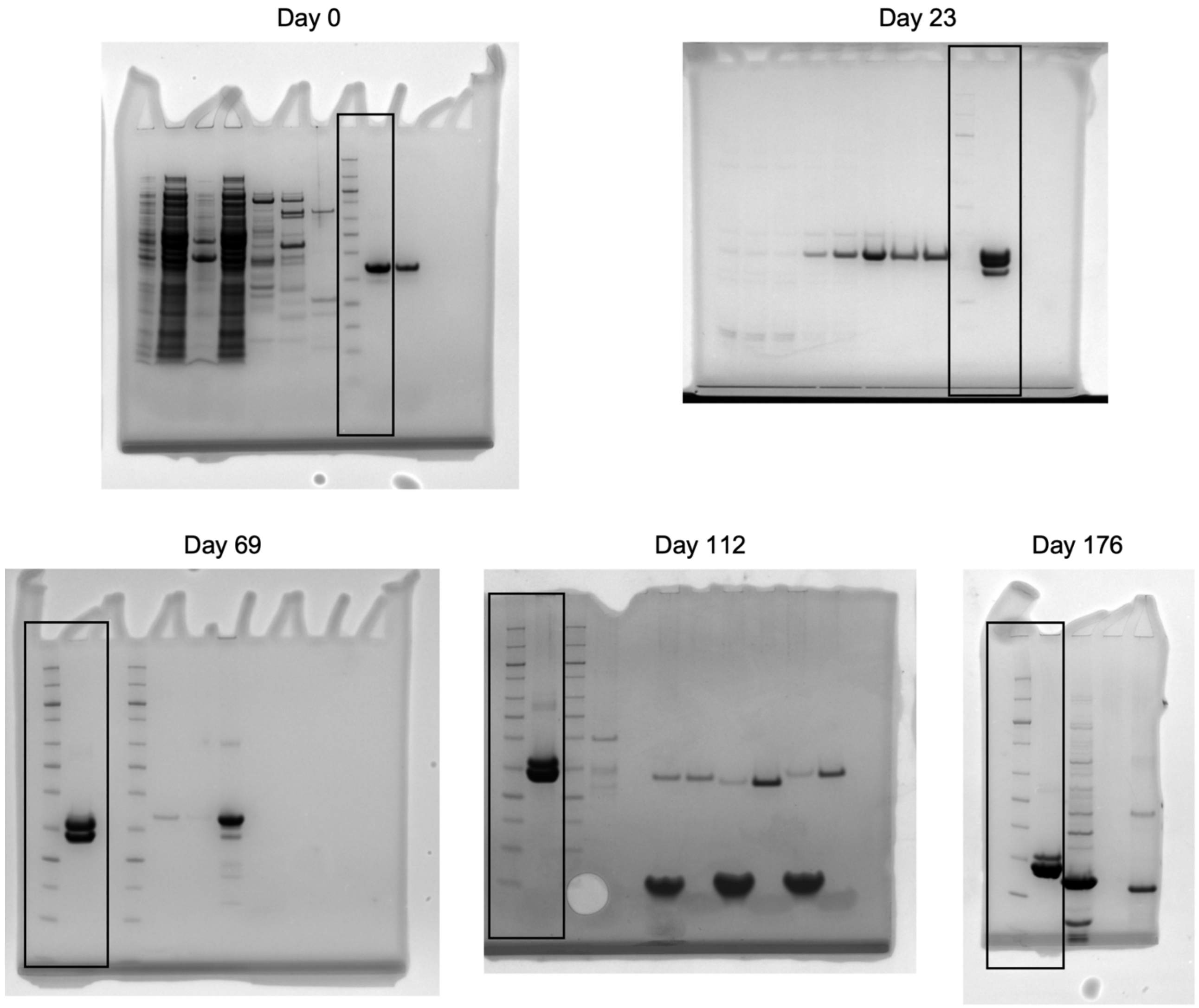

Supplementary Figure 25: Uncropped SDS-PAGE gels of PVA at different incubation times at 4ºC, related to Supplementary Fig. 4f. Black frames mark parts of gels shown in Supplementary Fig. 4f.

Panel c – ^{PepMoV}VLP (P), ^{TEV}VLP (P), ^{JGMV}VLP (P)

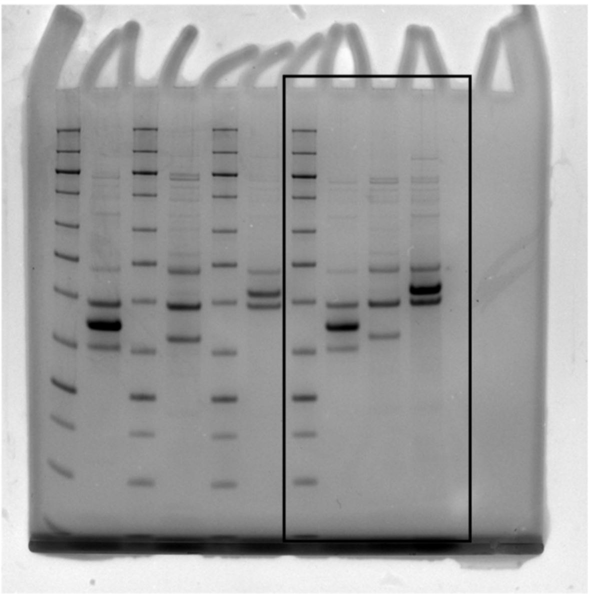

Panel e – ^{TEV}VLP (L)

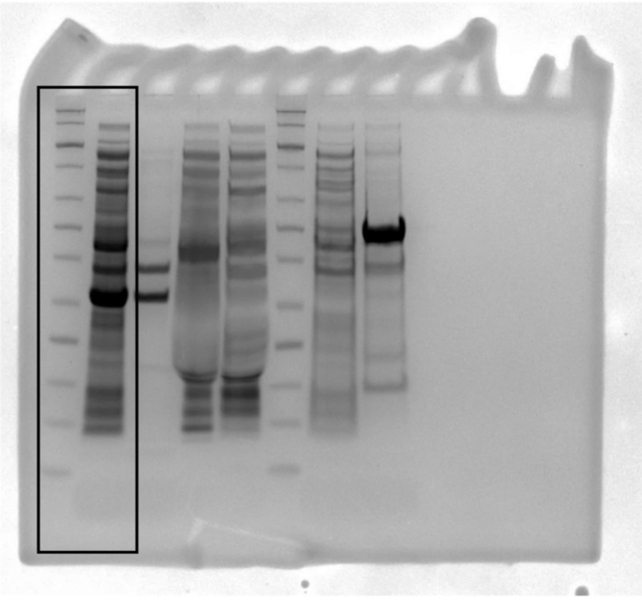

Supplementary Figure 26: Uncropped SDS-PAGE gels of ^{PepMoV}VLP (P), ^{TEV}VLP (P), ^{JGMV}VLP (P), and ^{TEV}VLP (L), related to Supplementary Fig. 13c, e. Black frames mark parts of gels shown in Supplementary Fig. 13, panels c and e.

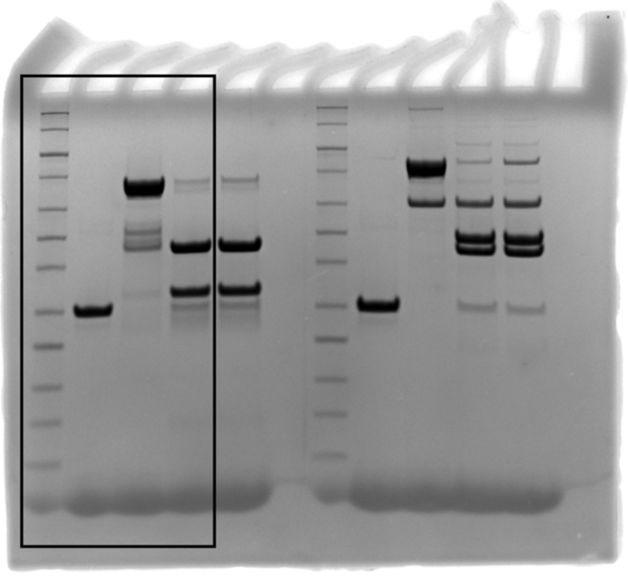

Supplementary Figure 27: Uncropped SDS-PAGE gel of ^{PVA}CP-MBP samples before and after TEV protease cleavage, related to Supplementary Fig. 16b. The black frame marks the part of the gel shown in Supplementary Fig. 16b.

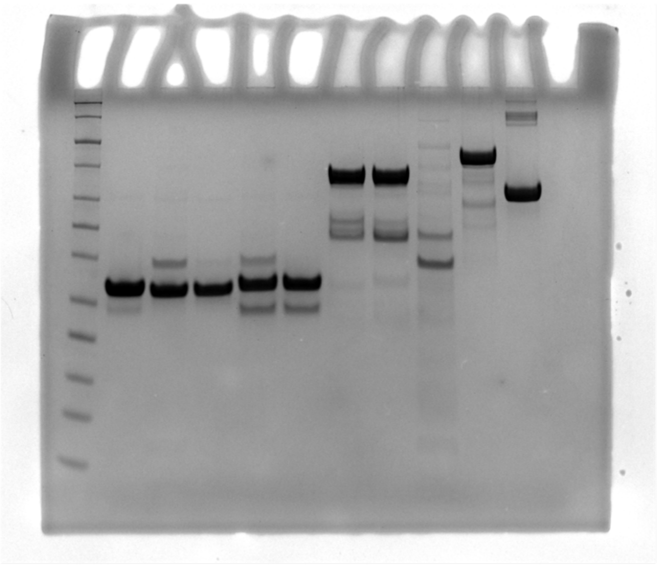

Supplementary Figure 28: Uncropped SDS-PAGE gel of samples for ATPase assay, related to Supplementary Fig. 17a.

Supplementary Table 1: VLP nomenclature.

	Filament architecture descriptors										
virus/VLP	h+RNA	h7.5	h8.5	h8.6	h8.7	h9.6	h9.7	h10.7	r8	r9	r10
PVA	x										
$^{\text{PVA-B11}}$VLP	x		x								
$^{\text{PVA-Datura}}$VLP	x		x								
^{PVA}VLP$^{\text{mut5}}$	x				x		x				x
^{PVA}VLP$^{\text{mut6}}$							x	x			x
^{PVA}VLP$^{\text{R163A}}$			x								
^{PVA}VLP$^{\text{D138C}}$	x										
^{PVA}VLP$^{\textit{in vitro}}$			x								
^{TEV}VLP			x								
^{PVY}VLP[5]	x	x							x		
^{PVY}VLP$^{\text{A161R}}$	x										
^{PVY}VLP$^{\text{Q87H}}$	x		x						x		
^{PVY}VLP$^{\text{R208T}}$	x	x	x						x		
^{PVY}VLP$^{\text{Q87H+R208T}}$	x		x								
^{PepMoV}VLP	x									x	
^{JGMV}VLP	x			x		x				x	

VLP$^{\text{h+RNA}}$...RNA-containing helical VLP
VLP^{hX}...RNA-free helical VLP
VLP^{rY}...RNA-free stacked-ring VLP

hX: X...number of subunits per helical turn
rY: Y...number of subunits per ring
h+RNA: in all cases 8.8 subunits per helical turn

'5' refers to reference number 5
'X' marks architectural types found in virus/VLP samples in this study. The nomenclature alone does not provide complete information about the filament architecture (helical symmetry). For details about helical symmetry, see Table 1.

Supplementary Table 2: Mass spectrometry analysis of the 40-kDa protein band in the SDS-PAGE gel of the ^{PVA}VLP sample (see Fig. 6e).

Protein name	Protein accession numbers	Alternate IDs	Protein molecular weight (Da)	Protein identification probability	Exclusive unique peptide count	Exclusive unique spectrum count	Exclusive spectrum count	Share of total spectra	Percentage sequence coverage	Length
Outer membrane porin F OS=Escherichia coli (strain K12) OX=83333 GN=ompF PE=1 SV=1	P02931	ompF	39334.4	100.0 %	30	94	1436	0.0643	90.1	362
Outer membrane protein assembly factor BamC OS=Escherichia coli (strain K12) OX=83333 GN=bamC PE=1 SV=1	P0A903	bamC	36842.3	100.0 %	15	22	57	0.00255	60.8	344
Outer membrane protein A OS=Escherichia coli (strain K12) OX=83333 GN=ompA PE=1 SV=1	P0A910, P0A911	ompA	37201	100.0 %	15	20	81	0.00363	58.4	346
Modulator of FtsH protease HflC OS=Escherichia coli (strain K12) OX=83333 GN=hflC PE=1 SV=1	P0ABC3, P0ABC4, P0ABC5	hflC	37650.9	100.0 %	16	19	45	0.00202	53.3	334
D-alanyl-D-alanine carboxypeptidase DacA OS=Escherichia coli (strain K12) OX=83333 GN=dacA PE=1 SV=1	P0AEB2, P0AEB3, P0AEB4	dacA	44445.5	100.0 %	12	12	18	0.000806	38.7	403
Outer membrane protein assembly factor BamB OS=Escherichia coli (strain K12) OX=83333 GN=bamB PE=1 SV=1	P77774	bamB	41886.9	100.0 %	11	15	45	0.00202	37.5	392
Maltose/maltodextrin-binding periplasmic protein OS=Escherichia coli (strain K12) OX=83333 GN=malE PE=1 SV=1	P0AEX9, P0AEY0	malE	43389.9	100.0 %	11	11	18	0.000806	35.4	396
Protein YdgH OS=Escherichia coli (strain K12) OX=83333 GN=ydgH PE=1 SV=1	P76177	ydgH	33903.3	100.0 %	5	5	5	0.000224	22.6	314
Cytochrome bd-I ubiquinol oxidase subunit 1 OS=Escherichia coli (strain K12) OX=83333 GN=cydA PE=1 SV=1	P0ABJ9, P0ABK0	cydA	58207,4	100.0 %	8	11	27	0.00121	19.5	522
Chain length determinant protein OS=Escherichia coli (strain K12) OX=83333 GN=wzzB PE=1 SV=2	P76372	wzzB	36455.6	100.0 %	3	3	4	0.000179	18.1	326
L-lactate dehydrogenase OS=Escherichia coli O9:H4 (strain HS) OX=331112 GN=lldD PE=3 SV=1	A8A670, B1IZI5, B1X8M0, B7NER0, B7NPB4, B7ULG1, C4ZXJ7, P33232, Q0TBK1, Q8FCB1	lldD	42729.8	100.0 %	6	6	11	0.000493	16.2	396
PTS system trehalose-specific EIIBC component OS=Escherichia coli (strain K12) OX=83333 GN=treB PE=1 SV=4	P36672	treB	51082.6	100.0 %	7	7	15	0.000672	15.0	473
Protein HemY OS=Escherichia coli (strain K12) OX=83333 GN=hemY PE=1 SV=1	P0ACB7, P0ACB8, P0ACB9	hemY	45246	100.0 %	4	4	6	0.000269	13.1	398
ECA polysaccharide chain length modulation protein OS=Escherichia coli (strain K12) OX=83333 GN=wzzE PE=1 SV=2	P0AG00, P0AG01	wzzE	39490.5	100.0 %	2	2	2	8.96E-05	9.8	348
Protein RecA OS=Escherichia coli O139:H28 (strain E24377A / ETEC) OX=331111 GN=recA PE=3 SV=1	A7ZQC8, A8A3H6, B1IUY0, B1LQ17, B5Z2B0, B6I685, B7LEB1, B7M9D6, B7MKG8, B7MYJ9, B7N6S9, B7NSH9, B7UHB7, C4ZYU4, P0A7G6, P0A7G7, P0A7G8, Q0TEI1, Q1R801	recA	37974.7	100.0 %	2	2	2	8.96E-05	8.5	353
Periplasmic serine endoprotease DegP OS=Escherichia coli (strain K12) OX=83333 GN=degP PE=1 SV=1	P0C0V0, P0C0V1	degP	49354.9	100.0 %	3	3	3	0.000134	8.2	474
DNA-directed RNA polymerase subunit alpha OS=Escherichia coli O1:K1 / APEC OX=405955 GN=rpoA PE=3 SV=1	A1AGI6, A7ZSI4, A8A5A0, P0A7Z4, P0A7Z5, P0A7Z6, Q0TCG6, Q1R637	rpoA	36512.1	100.0 %	3	3	5	0.000224	7.6	329
Membrane-bound lytic murein transglycosylase A OS=Escherichia coli (strain K12) OX=83333 GN=mltA PE=1 SV=1	P0A935, P0A936	mltA	40411.2	100.0 %	2	2	2	8.96E-05	7.4	365
Oligopeptide transport ATP-binding protein OppF OS=Escherichia coli (strain K12) OX=83333 GN=oppF PE=3 SV=1	P77737	oppF	37198.1	100.0 %	2	2	3	0.000134	6.3	334
Sensor protein BasS OS=Escherichia coli (strain K12) OX=83333 GN=basS PE=1 SV=1	P30844	basS	41030.3	100.0 %	2	2	2	8.96E-05	6.1	363
Maltose operon periplasmic protein OS=Escherichia coli (strain K12) OX=83333 GN=malM PE=3 SV=1	P03841	malM	31943.9	100.0 %	2	2	3	0.000134	5.6	306
Oligopeptide transport ATP-binding protein OppD OS=Escherichia coli (strain K12) OX=83333 GN=oppD PE=1 SV=2	P76027	oppD	37189.2	100.0 %	2	3	3	0.000134	5.3	337
Maltose/maltodextrin import ATP-binding protein MalK OS=Escherichia coli (strain K12) OX=83333 GN=malK PE=1 SV=1	P68187, P68188, Q1R3Q1, Q8FB37	malK	40989.9	100.0 %	2	2	3	0.000134	5.1	371
Modulator of FtsH protease HflK OS=Escherichia coli (strain K12) OX=83333 GN=hflK PE=1 SV=1	P0ABC7, P0ABC8	hflK	45545.5	100.0 %	2	2	3	0.000134	5.0	419
Uncharacterized protein YaiW OS=Escherichia coli (strain K12) OX=83333 GN=yaiW PE=4 SV=1	P77562	yaiW	40415.5	100.0 %	2	2	3	0.000134	5.0	364
NAD(P) transhydrogenase subunit beta OS=Escherichia coli (strain K12) OX=83333 GN=pntB PE=1 SV=1	P0AB67, P0AB68, P0AB69	pntB	48724	100.0 %	2	2	2	8.96E-05	4.3	462

Supplementary Table 3: List of mutagenic oligonucleotides.

Target	Mutation	Oligonucleotide sequence (5' → 3')
^{PVA}CP	H89Q	TGCTAGAGCCACCCAGGAACAATTCCAAAAC
	E90S	CTAGAGCCACCCACTCACAATTCCAAAACTG
	D138C	TTTGGACCATGATGTGTAATGAGGAACAAGT
	S144E	AATGAGGAACAAGTGGAATATCCATTAAAACCC
	R163A	TTAAGGCAAATTATGGCACATTTCAGCGCACTC
	K180E	ATGAGAAGTCGTGAGGAACCATACATGCCCA
	T210R	TATGAGATCACTGCACGCACTCCGATCAGAGCC
^{PVA}CP^{E90S}	H89Q	TGCTAGAGCCACCCAGTCACAATTCCAAAAC
^{PVA}CP^{H89Q}	E90S	CTAGAGCCACCCAGTCACAATTCCAAAACTG
^{PVY}CP	Q87H	AATACTCGAGCAACTCATTCACAGTTTGATACG
	E142S	GGAGATGAACAAGTCTCATACCCACTGAAACCA
	A161R	CTTAGGCAAATCATGCGCCATTTCTCAGATGTT
	R208T	TATGAGGTCACATCAACAACACCAGTGAGGGCT

^{PVA}CP = $^{PVA\text{-}Datura}$CP

Supplementary References

1. Crooks, G. E., Hon, G., Chandonia, J. M. & Brenner, S. E. WebLogo: A sequence logo generator. *Genome Res* **14**, 1188–1190 (2004).
2. Edgar, R. C. MUSCLE: Multiple sequence alignment with high accuracy and high throughput. *Nucleic Acids Res* **32**, 1792–1797 (2004).
3. Eskelin, K., Suntio, T., Hyvärinen, S., Hafren, A. & Mäkinen, K. Renilla luciferase-based quantitation of Potato virus A infection initiated with Agrobacterium infiltration of N. benthamiana leaves. *J Virol Methods* **164**, 101–110 (2010).
4. De, S. *et al.* Potato virus A particles – A versatile material for self-assembled nanopatterned surfaces. *Virology* **578**, 103–110 (2023).
5. Kavčič, L. *et al.* From structural polymorphism to structural metamorphosis of the coat protein of flexuous filamentous potato virus Y. *Commun Chem* **7**, 1–19 (2024).
6. Kežar, A. *et al.* Structural basis for the multitasking nature of the potato virus Y coat protein. *Sci Adv* **5**, eaaw3808 (2019).
7. Zamora, M. *et al.* Potyvirus virion structure shows conserved protein fold and RNA binding site in ssRNA viruses. *Sci Adv* **3**, eaao2182 (2017).
8. Cuesta, R. *et al.* Structure of Turnip mosaic virus and its viral-like particles. *Sci Rep* **9**, 15396 (2019).
9. Dolinsky, T. J., Nielsen, J. E., McCammon, J. A. & Baker, N. A. PDB2PQR: An automated pipeline for the setup of Poisson-Boltzmann electrostatics calculations. *Nucleic Acids Res* **32**, W665–W667 (2004).
10. Baker, N. A., Sept, D., Joseph, S., Holst, M. J. & Andrew McCammon, J. Electrostatics of nanosystems: Application to microtubules and the ribosome. *Proc Natl Acad Sci U S A* **98**, 10037–10041 (2001).
11. GraphPad Software. GraphPad Prism. Preprint at (2024).
12. Norby, J. G. Coupled assay of Na+,K+-ATPase activity. *Methods Enzymol* **156**, 116–119 (1988).
13. Estrozi, L. F., Desfosses, A. & Schoehn, G. Helixplorer-1: Online Indexation of Fibers and Helical Structures. *Rico.ibs.fr* (2018).
