# Supplementary material for: Species-specific structural adaptation of the potyviral coat protein in virions and virus-like particles
Source: Commun Biol. 2026 Jan 13;9:226. doi: 10.1038/s42003-025-09502-w (PMC12902108; doi:10.1038/s42003-025-09502-w)
Supplement: Supplementary file 3 — Description of Additional Supplementary Files [file 42003_2025_9502_MOESM3_ESM.pdf]

Description of Additional Supplementary Files

File name: Supplementary Data 1
Description: The source data behind the graphs in the paper.

File name: Supplementary Data 2
Description: Initial and final helical parameters of all filamentous structures from this study.
