# Supplementary material for: Species-specific structural adaptation of the potyviral coat protein in virions and virus-like particles
Source: Commun Biol. 2026 Jan 13;9:226. doi: 10.1038/s42003-025-09502-w (PMC12902108; doi:10.1038/s42003-025-09502-w)
Supplement: Supplementary file 6 — Reporting Summary [file 42003_2025_9502_MOESM6_ESM.pdf]

nature portfolio

Corresponding author(s): Kristiina Mäkinen, Marjetka Podobnik

Last updated by author(s): Dec 22, 2025

Reporting Summary

Nature Portfolio wishes to improve the reproducibility of the work that we publish. This form provides structure for consistency and transparency in reporting. For further information on Nature Portfolio policies, see our Editorial Policies and the Editorial Policy Checklist.

Statistics

For all statistical analyses, confirm that the following items are present in the figure legend, table legend, main text, or Methods section.

n/a	Confirmed	
☐	☒	The exact sample size (*n*) for each experimental group/condition, given as a discrete number and unit of measurement
☐	☒	A statement on whether measurements were taken from distinct samples or whether the same sample was measured repeatedly
☐	☒	The statistical test(s) used AND whether they are one- or two-sided<br>*Only common tests should be described solely by name; describe more complex techniques in the Methods section.*
☒	☐	A description of all covariates tested
☐	☒	A description of any assumptions or corrections, such as tests of normality and adjustment for multiple comparisons
☐	☒	A full description of the statistical parameters including central tendency (e.g. means) or other basic estimates (e.g. regression coefficient) AND variation (e.g. standard deviation) or associated estimates of uncertainty (e.g. confidence intervals)
☐	☒	For null hypothesis testing, the test statistic (e.g. *F*, *t*, *r*) with confidence intervals, effect sizes, degrees of freedom and *P* value noted<br>*Give P values as exact values whenever suitable.*
☒	☐	For Bayesian analysis, information on the choice of priors and Markov chain Monte Carlo settings
☒	☐	For hierarchical and complex designs, identification of the appropriate level for tests and full reporting of outcomes
☒	☐	Estimates of effect sizes (e.g. Cohen's *d*, Pearson's *r*), indicating how they were calculated

Our web collection on statistics for biologists contains articles on many of the points above.

Software and code

Policy information about availability of computer code

Data collection	No custom code was used for data collection, all other commercial and open source software is listed and referenced in the text (incl. version number).
Data analysis	No custom code was used for data analysis, all other commercial and open source software is listed and referenced in the text and also copied below (incl. version number):<br><br>Software used: OriginPro2018 v8.1, GraphPad Prism v10.2, SPSS Statistics v29.0.2.0, Digital Micrograph software v2.1.1, Velox v3.0, Mascot v2.8.3, cryoSPARC v4.3, ChimeraX v1.8, PyMOL v2.5.5, Coot v0.9.4.1, Phenix v1.20.1

For manuscripts utilizing custom algorithms or software that are central to the research but not yet described in published literature, software must be made available to editors and reviewers. We strongly encourage code deposition in a community repository (e.g. GitHub). See the Nature Portfolio guidelines for submitting code & software for further information.

Data

Policy information about availability of data

All manuscripts must include a data availability statement. This statement should provide the following information, where applicable:

- Accession codes, unique identifiers, or web links for publicly available datasets
- A description of any restrictions on data availability
- For clinical datasets or third party data, please ensure that the statement adheres to our policy

Cryo-EM maps and atomic models have been deposited in the Electron Microscopy Data Bank (EMDB) and wwPDB, respectively, with EMDB/PDB accession codes: EMD-53790/9R7R, EMD-53791/9R7S, EMD-53792/9R7T, EMD-53793/9R7U, EMD-53794/9R7V, EMD-53796/9R7X, EMD-53799/9R7Y, EMD-53800/9R7Z, EMD-53801/9R80, EMD-53802/9R81, EMD-53862/9R9W, EMD-53863/9R9X, EMD-53864/9R9Y, EMD-53865/9R9Z, EMD-53866/9RA0, EMD-53867/9RA1, EMD-53868/9RA2, EMD-53632, EMD-53633, EMD-53634, EMD-53635, EMD-53636, EMD-53638, EMD-53639, EMD-53640, EMD-53642, EMD-53643, EMD-53644, EMD-53645, EMD-53646, EMD-53647, EMD-53650, and EMD-53651, with corresponding structures and atomic models provided in Table 1. Raw cryo-EM datasets have been deposited to the Electron Microscopy Public Image Archive (EMPIAR) with accession codes EMPIAR-12819 (EMD-53790/9R7R), EMPIAR-12820 (EMD-53791/9R7S), EMPIAR-12821 (EMD-53792/9R7T, EMD-53793/9R7U), EMPIAR-12822 (EMD-53794/9R7V, EMD-53796/9R7X), EMPIAR-12823 (EMD-53799/9R7Y, EMD-53800/9R7Z, EMD-53801/9R80), EMPIAR-12824 (EMD-53802/9R81), EMPIAR-12825 (EMD-53862/9R9W), EMPIAR-12826 (EMD-53863/9R9X, EMD-53864/9R9Y), EMPIAR-12827 (EMD-53865/9R9Z, EMD-53866/9RA0, EMD-53867/9RA1, EMD-53868/9RA2), EMPIAR-12828 (EMD-53632, EMD-53633, EMD-53634, EMD-53635), EMPIAR-12829 (EMD-53636), EMPIAR-12830 (EMD-53638, EMD-53639, EMD-53640), EMPIAR-12831 (EMD-53642, EMD-53643, EMD-53644, EMD-53645), EMPIAR-12832 (EMD-53646, EMD-53647), EMPIAR-12833 (EMD-53650), and EMPIAR-12834 (EMD-53651). All data are available in the main text, figures, tables, and Supplementary Information file. Source data for all graphs are provided in the Supplementary Data 1 file. Initial and final helical parameters of all filamentous structures from this study are provided in the Supplementary Data 2 file. Uncropped images of gels, supporting Fig. 6e and Supplementary Figs. 2a, 4f, 13c, 13e, 16b and 17a are shown in the Supplementary Information in Supplementary Figs. 23-28, respectively. Other data related to this paper may be requested from the authors.

Research involving human participants, their data, or biological material

Policy information about studies with human participants or human data. See also policy information about sex, gender (identity/presentation), and sexual orientation and race, ethnicity and racism.

Reporting on sex and gender	n/a
Reporting on race, ethnicity, or other socially relevant groupings	n/a
Population characteristics	n/a
Recruitment	n/a
Ethics oversight	n/a

Note that full information on the approval of the study protocol must also be provided in the manuscript.

Field-specific reporting

Please select the one below that is the best fit for your research. If you are not sure, read the appropriate sections before making your selection.

☒ Life sciences ☐ Behavioural & social sciences ☐ Ecological, evolutionary & environmental sciences

For a reference copy of the document with all sections, see nature.com/documents/nr-reporting-summary-flat.pdf

Life sciences study design

All studies must disclose on these points even when the disclosure is negative.

Sample size	For all experiments requiring statistical analysis, we aimed to have a minimum of n = 3 replicates in order to derive mean and p-values. Exact sample sizes are provided either in the Method section or in the Figure legends.
Data exclusions	No data was excluded.
Replication	All measurements were replicated at least 3 times. Exact numbers of replicates are stated either in the Method section or in the Figure legends. Data replication was successful.
Randomization	n/a
Blinding	n/a

Reporting for specific materials, systems and methods

We require information from authors about some types of materials, experimental systems and methods used in many studies. Here, indicate whether each material, system or method listed is relevant to your study. If you are not sure if a list item applies to your research, read the appropriate section before selecting a response.

Materials & experimental systems

n/a	Involved in the study
☐	☒ Antibodies
☒	☐ Eukaryotic cell lines
☒	☐ Palaeontology and archaeology
☒	☐ Animals and other organisms
☒	☐ Clinical data
☒	☐ Dual use research of concern
☐	☒ Plants

Methods

n/a	Involved in the study
☒	☐ ChIP-seq
☒	☐ Flow cytometry
☒	☐ MRI-based neuroimaging

Antibodies

Antibodies used: anti-PVA CP antibodies: PVAmix mAb, SASA (Science and Advice for Scottish Agriculture), batches 24, 63, 66

Validation: *Describe the validation of each primary antibody for the species and application, noting any validation statements on the manufacturer's website, relevant citations, antibody profiles in online databases, or data provided in the manuscript.*

Plants

Seed stocks: Nicotiana benthamiana (Lab strain) Ref. Bally et al. (2018) Annu Rev Phytopathol. 25;56:405-426. doi: 10.1146/annurev-phyto-080417-050141.

Novel plant genotypes: *Describe the methods by which all novel plant genotypes were produced. This includes those generated by transgenic approaches, gene editing, chemical/radiation-based mutagenesis and hybridization. For transgenic lines, describe the transformation method, the number of independent lines analyzed and the generation upon which experiments were performed. For gene-edited lines, describe the editor used, the endogenous sequence targeted for editing, the targeting guide RNA sequence (if applicable) and how the editor was applied.*

Authentication: *Describe any authentication procedures for each seed stock used or novel genotype generated. Describe any experiments used to assess the effect of a mutation and, where applicable, how potential secondary effects (e.g. second site T-DNA insertions, mosiacism, off-target gene editing) were examined.*
